# Cooperative interaction among BMAL1, HSF1, and p53 protects mammalian cells from UV stress

**DOI:** 10.1038/s42003-018-0209-1

**Published:** 2018-11-22

**Authors:** Genki Kawamura, Mitsuru Hattori, Ken Takamatsu, Teruyo Tsukada, Yasuharu Ninomiya, Ivor Benjamin, Paolo Sassone-Corsi, Takeaki Ozawa, Teruya Tamaru

**Affiliations:** 1 Department of Chemistry, School of Science, The University of Tokyo, 7-3-1 Hongo, Bunkyo-ku, Tokyo, 133-0033 Japan; 2 Department of Physiology & Advanced Research Center for Medical Science, Toho University School of Medicine, 5-21-16 Omori-nishi, Ota-ku Tokyo, 143-8540 Japan; 3 Nishina Center for Accelerator-Based Science, Riken, 2-1 Hirosawa, Wako Saitama, 351-0198 Japan; 4 Department of Radiation Effects Research, National Institute of Radiological Sciences, National Institutes for Quantum and Radiological Science and Technology, 4-9-1 Anagawa, Inage-ku, Chiba-shi Chiba, 263-8555 Japan; 5 Department of Medicine, Froedtert & Medical College of Wisconsin, 8701 Watertown Plank Rd., Milwaukee, WI 53226 USA; 6 Center for Epigenetics and Metabolism, School of Medicine, University of California Irvine, California, 92697 USA

## Abstract

The circadian clock allows physiological systems to adapt to their changing environment by synchronizing their timings in response to external stimuli. Previously, we reported clock-controlled adaptive responses to heat-shock and oxidative stress and showed how the circadian clock interacts with BMAL1 and HSF1. Here, we present a similar clock-controlled adaptation to UV damage. In response to UV irradiation, HSF1 and tumor suppressor p53 regulate the expression of the clock gene *Per2* in a time-dependent manner. UV irradiation first activates the HSF1 pathway, which subsequently activates the p53 pathway. Importantly, BMAL1 regulates both HSF1 and p53 through the BMAL1–HSF1 interaction to synchronize the cellular clock. Based on these findings and transcriptome analysis, we propose that the circadian clock protects cells against the UV stress through sequential and hierarchical interactions between the circadian clock, the heat shock response, and a tumor suppressive mechanism.

## Abstract (web summary)

Genki Kawamura et al. demonstrate that cells are protected against UV stress through cooperative interactions among circadian clock, heat shock response, and a tumor suppression mechanism. This study reports another protective role of circadian clock as an adaptation strategy against cellular stress.

## Introduction

The circadian clock is a cell-autonomous timing system that oscillates with ~24 h periodicity and regulates global gene expression in almost every cell in the body. The cellular clock can synchronize its daily phase in response to external factors, including growth factors and stress stimulation, maintaining cellular homeostasis and eliciting cellular adaptation to the surrounding environment^1,2^.

To understand the interplay between circadian clock components and stress response systems, a molecular-level understanding of the adaptation process, including circadian clock synchronization, is crucial. In mammals, clock oscillation is produced through positive and negative transcriptional–translational feedback loops driven by circadian transcription factors such as brain and muscle ARNT-like protein-1 (BMAL1) and Circadian Locomotor Output Cycles Kaput (CLOCK). The BMAL1:CLOCK heterodimer binds to the E-box and transactivates the core clock genes *Period* (*Per*) and *Cryptochrome* (*Cry*) and genome-wide clock-controlled genes. PER2 periodically forms a heterodimer with PER1, CRY1, or CRY2 by binding to their respective PAS domains. After nuclear translocation, PER1-2 and CRY1-2 associate with BMAL1:CLOCK to inhibit *Per1-2* and *Cry1-2* expression^3,4^. Activation of BMAL1 transcriptional activity and/or regulation of clock genes initiates clock synchronization by modulating the molecular clock.

Previously, we attempted to elucidate how the clock system contributes to the cellular stress-responsive protection system. We found that cell stress by heat shock or reactive oxygen species (ROS) triggers the synchronization of the circadian clock via interactions between BMAL1 and heat-shock factor-1 (HSF1), a central transcription factor for the heat-shock response (HSR) pathway. The synchronized clock, in turn, regulates the expression of stress resistance genes via activation of the HSR pathway and various adaptive protection pathways that control anti-oxidant and cell survival responses to protect cells from the stressors^5–7^. The concept of a “circadian adaptation system”, in which the clock system functions as a platform to invoke daily time-dependent adaptive responses through its interplay with various stress protection systems, has become increasingly clear. Several groups have also identified the clock-driven stress protection system that is regulated by the interplay between clock components and HSF1 or the tumor suppressor p53^8,9^. Furthermore, in the p53-mediated anti-genotoxic response, *Cry1-2* and *Per2* both affect the sensitivity of p53 to stresses^10,11^. Recent findings have suggested that the anti-genotoxic pathway may be affected by HSF1, leading to the possibility that the anti-genotoxic and HSR pathways act cooperatively for cellular protection^12,13^. Because numerous stress-responsive genes are clock-controlled genes, the molecular clock is essential for cell survival after critical damage^14^. Indeed, disruption of clock components leads to altered sensitivity to cell stress, resulting in proliferation disorders and tumor progression^15,16^.

To understand clock-dependent stress adaptation, analysis of circadian time (CT) dependency is necessary. Here, we examined CT-dependent molecular processes, specifically at the early stage, in response to genotoxic damage induced by UV light irradiation. We used mouse embryonic fibroblast (MEF) cell lines, including NIH3T3 cells, wild-type MEFs, BMAL1-deficient MEFs^17^, HSF1-deficient MEFs^18^, and p53-deficient MEFs^19^, and human osteosarcoma U2OS cells, as models for the circadian clock study. First, we investigated which transcription factors were activated by UV irradiation and involved in clock synchronization, and found that in addition to HSF1, p53 was involved in the synchronization. Second, we analyzed the interplay between the clock, HSR and tumor suppressor system and found that these components directly interacted with each other and therefore, trans-activation of the stress response factors mutually affected their functions. Finally, to elucidate CT-dependent networking between the clock, HSR, and tumor suppressor system, we assessed the CT-dependent behaviors of their responsible pathways during UV-triggered clock synchronization.

## Results

### UV irradiation synchronizes circadian clocks via the HSR

**Fig. 1:**
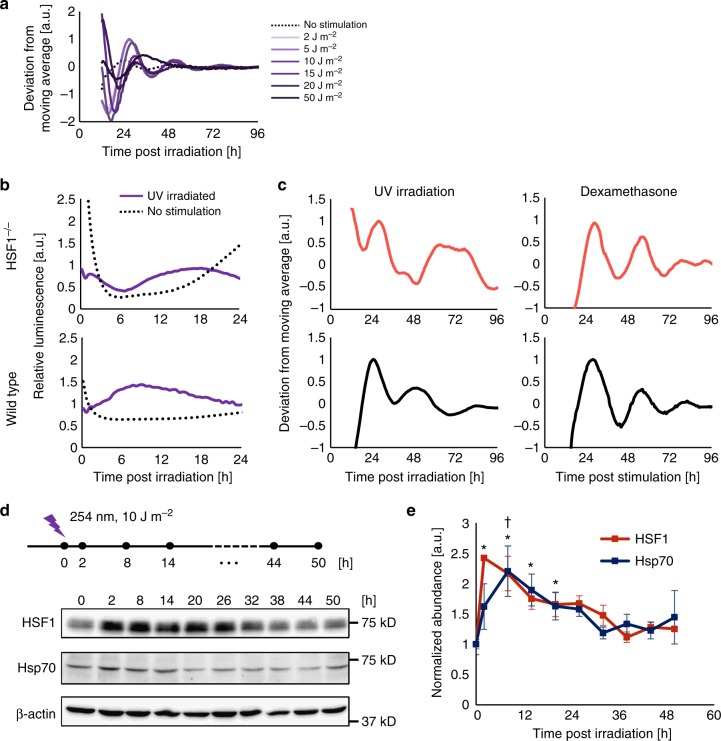
HSF1 is essential for circadian clock synchronization by UV irradiation. **a** NIH3T3 cells harboring Per2-Luc were irradiated with different doses of UV light (254 nm), and the temporal luminescence profile after the irradiation was monitored. Representative Per2-Luc profiles with normalized values deduced from the deviation from the moving average values are shown. *n* = 3. **b**, **c** Wild-type and HSF1−/− MEFs harboring the Per2-Luc reporter were stimulated by UV irradiation (254 nm, 10 J m^−2^). Per2-Luc profiles were monitored by real-time bioluminescence assay. Relative luminescence intensity of the initial response after UV irradiation (**b**). Circadian profiles illustrated by plotting the detrended values are shown for UV-irradiated (254 nm, 10 J m^−2^) and Dex (dexamethasone)-treated (10 nM, 2 h) cells (**c**). **d**, **e** Expression of HSR proteins, HSF1 and Hsp70, after UV irradiation (10 J m^−2^) by western blotting and quantification. Representative blot of three independent experiments (**d**, *n* = 3) and a graph plotting normalized protein expression (**e**). See Supplementary Figure 13 for images of full blots. *p < 0.05 for HSF1, ^†^*p* < 0.05 for Hsp70; not significant (*p* > 0.05) unless mentioned, error bar: SD

First, we evaluated the optimal dose of UV irradiation that synchronizes the circadian clock to create a model for the genotoxic stress response of the mammalian cellular clock. To this end, NIH3T3 fibroblasts harboring the *Per2* promoter-driven firefly luciferase (Per2-Luc) reporter were used to analyze temporal Per2-Luc profiles in living cells post irradiation with various strengths of UV stimulation (254 nm). A dose-dependent surge in Per2-Luc luminescence after UV irradiation was observed with 2 to 30 J m^−2^, and the peak intensity decreased at doses over 50 J m^−2^, indicating that Per2-Luc responded to genotoxic stress in a dose-dependent manner before reaching a critical dose (30 J m^−2^), when cell viability substantially dropped (Supplementary Figure 1a–c). Additionally, a rhythmic Per2-Luc pattern was observed at 2 to 20 J m^−2^ and was most evident at 10 J m^−2^, indicating that cell stress caused by UV irradiation with the appropriate dose triggered clock synchronization (Fig. 1a and Table 1). Interestingly, we found a weak negative correlation between period length and amplitude; cells with large a Per2-Luc amplitude have a shorter period length (Supplementary Figure 1d). We reasoned that Per2-Luc upregulation at the early stage during the synchronization may shorten period length. To examine circadian fluctuations in clock gene expression, we quantified the expression profiles of core clock genes, namely, *Bmal1*, *Clock*, *Per1-3*, and *Cry1-2* after 10 J m^−2^ UV irradiation (Supplementary Figure 2). Circadian fluctuation of all endogenous clock genes verified UV-triggered clock synchronization. Importantly, an acute surge of endogenous *Per2* expression was observed, suggesting that modulation of *Per2* is a key regulatory step for the synchronization. Based on these data, we used 10 J m^−2^ as the appropriate dose for genotoxic stimulation to induce a circadian response and the cellular protection system.

**Table 1:** The period calculated from each of the Per2-Luc profiles using the Cosinor program

Stimulation	Period [h]
2 J m^−2^	25.3 ± 0.5
5 J m^−2^	25.3 ± 0.8
10 J m^−2^	24.8 ± 0.7
15 J m^−2^	22.3 ± 1.2
20 J m^−2^	21.1 ± 1.9
30 J m^−2^	Not periodic
50 J m^−2^	Not periodic
100 J m^−2^	Not periodic

Based on our recent findings that the HSR pathway is responsible for the clock synchronization triggered by heat-shock- and ROS-induced cell stress^6,7^, we investigated the role of HSR in UV-triggered clock synchronization. In our previously presented model, HSF1, a central transcription factor of HSR, mediates circadian clock reset via induction of *Per2* expression. Thus, we analyzed the Per2-Luc reporter response in wild-type and HSF1-dficient MEFs after UV irradiation. Per2-Luc began to elevate within 6 h post irradiation and then exhibited a circadian oscillation in the wild-type cells; however, in HSF1−/− cells, no apparent initial surge or circadian oscillation of Per2-Luc was observed (Fig. 1b, c). This observation demonstrates that the HSF1-controlled HSR is essential for UV-triggered clock synchronization and functions by inducing a synchronous *Per2* surge after UV stimulation. Interestingly, dexamethasone (Dex) treatment synchronized both wild-type and HSF1−/− cells. The failure of HSF1−/− cells to synchronize in response to stress stimulation was consistent with the findings of previous reports^6,7^, which strongly suggests that HSF1 is commonly necessary for cell stress-triggered clock synchronization.

We next investigated whether the HSR is activated upon UV irradiation. To this end, we analyzed HSR activation by quantification of the change in the abundance of HSF1 trans-activated gene products after stimulation (Fig. 1d, e). The protein abundance of the HSR products, namely, HSF1 and heat-shock protein 70 (Hsp70), acutely increased within 2 h post irradiation, suggesting that UV irradiation triggered HSR transcriptional activity. HSF1-mediated trans-activation was confirmed by quantification of the mRNA levels of HSR targets, namely, *Hsf1* and *Hsp70*^20^, after irradiation (Supplementary Figure 3a). *Hsf1* and *Hsp70* mRNA expression increased within 1 h after the irradiation, consistent with the increases in HSF1 and Hsp70 protein expression upon stimulation. Transcriptional activation of HSF1 was further confirmed by a reporter assay of a heat-shock response element (HSE) sequence, which is known to be bound by HSF1. We conducted single-cell imaging of the HSE-driven railroad worm red luciferase (HSE-SLR) reporter after UV irradiation (Supplementary Figure 3b). An acute increase in the HSE-SLR luminescence of single cells was observed, suggesting that HSF1 activity was evoked synchronously after irradiation. Because HSF1 activation occurred synchronously in each individual cell, this result indicates that HSF1 activation is critical for UV-triggered clock synchronization.

**Fig. 2:**
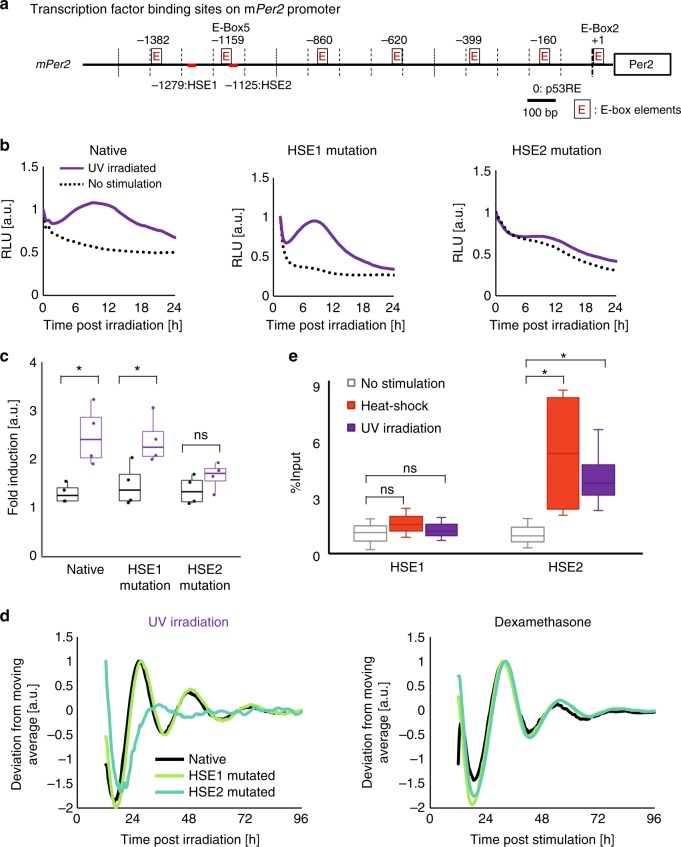
HSF1 binds to the *Per2* promoter during UV-triggered clock synchronization. **a** Schematic diagram of the mouse *Per2* promoter showing key transcription binding sites: E-boxes, HSEs and the p53RE. Numbers indicate the position relative to the translation start site. **b**, **c** Quantification of the Per2-Luc initial response after UV irradiation of cells expressing the native reporter Per2-Luc, HSE-1-mutated Per2-Luc reporter or HSE-2-mutated Per2-Luc reporter. NIH3T3 cells expressing either native Per2-Luc, HSE1 site-mutated Per2-Luc (HSE1-mut) or HSE2 site-mutated Per2-Luc (HSE2-mut) were treated with UV irradiation (10 J m^−2^), and the luminescence profiles after stimulation were monitored. Representative luminescence profiles are shown (**b**). Box plot corresponds to the relative change in the Per2-Luc response before and after irradiation (**c**). *n* = 4, *p < 0.05, ns: not significant (*p* > 0.05), two-tailed *t*-test. **d** Circadian luminescence profiles upon Dex (100 nM, 2 h) treatment or UV irradiation (10 J m^−2^) normalized to the maximum value are shown. **e** ChIP assay of HSE1 and HSE2 after UV irradiation (10 J m^−2^) and heat shock (43 °C, 30 min) using the anti-HSF1 antibody. Immunoprecipitated DNA fragments were quantified by qPCR. *n* = 3, *p < 0.05, ns: not significant (*p* > 0.05), two-tailed *t*-test

Because the mouse *Per2* promoter carries two HSE sites, namely, HSE1 and HSE2, adjacent to the heat-shock-responsive E-box^7^, we examined whether HSF1 also triggered synchronous circadian *Per2* expression after binding to the HSE sites post UV irradiation (Fig. 2a). To examine which HSE sites were required for UV-triggered clock reset, we analyzed the *Per*2-Luc profile with mutagenesis performed in either of the HSEs in the *Per2* promoter after UV exposure. NIH3T3 cells expressing native Per2-Luc or HSE-mutated Per2-Luc were stimulated with UV irradiation or Dex. The acute surge in native Per2-Luc after UV irradiation was dramatically impaired in the HSE2-mutated Per2-Luc cells, while HSE1-mutated Per2-Luc cells exhibited a clear surge upon stimulation (Fig. 2b, c). Importantly, the circadian profile of HSE2-mutated Per2-Luc showed an arrhythmic pattern, indicating that HSE2 is indispensable for UV-triggered clock synchronization (Fig. 2d). With Dex treatment, HSE mutagenesis did not affect circadian Per2-Luc profiles, as reported previously^7^. We further validated the origin of the acute surge using reporters originating from HSE sequences in the Per2-Luc reporter (minimal Per2HSE reporters), in which a luciferase is fused downstream of a native or mutated HSE sequence of the *Per2* promoter (Supplementary Figure 4). We observed less activation in the HSE2-mutated minimal Per2HSE reporter than in the unmutated reporter, consistent with the impaired acute surge in the HSE2-mutated Per2-Luc reporter. To assess the binding of HSF1 to HSE, a chromatin immunoprecipitation assay (ChIP assay) was performed after UV irradiation (Fig. 2e). We performed a ChIP assay at 2 h after stimulation to assess the acute HSF1 response to the UV irradiation and found that HSF1 bound dominantly to HSE2 but not to HSE1, as also observed with heat-shock-treated cells. These results demonstrated that UV irradiation triggers activation of HSR, inducing HSF1 binding to the HSE2 site on the *Per2* promoter, thereby enhancing *Per2* expression.

### p53 represses *Per2* expression during UV-mediated clock reset

**Fig. 3:**
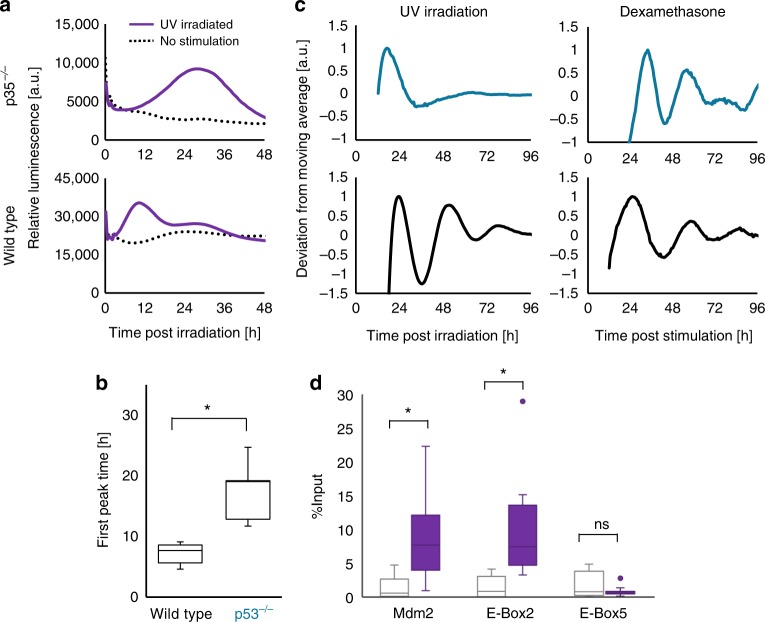
p53 activation is required for UV-triggered clock synchronization. **a**–**c** p53−/− MEFs harboring the Per2-Luc reporter were stimulated by UV irradiation (254 nm, 10 J m^−2^), and the temporal variation of Per2-Luc expression was monitored by bioluminescence analysis. Initial response of Per2-Luc between 0 and 48 h after UV irradiation (**a**). Comparison of the time at which the Per2-Luc intensity reached its maximum between wild-type and p53−/− MEFs (**b**). Circadian profiles are shown (*n* = 3) for UV-irradiated (254 nm, 10 J m^−2^) and Dex-treated (10 nM, 2 h) cells (**c**). **d** ChIP assay of the p53RE adjacent to E-Box2, the HSE adjacent to E-Box5 and the p53RE of the *Mdm*2 promoter after UV irradiation (10 J m^−2^) using the anti-p53 antibody. Immunoprecipitated DNA fragments were quantified by qPCR. *n* = 3, *p < 0.05, ns: not significant (*p* > 0.05), two-tailed *t*-test

Although HSF1 has been shown to be a transactivator of *Per2* post UV irradiation, according to the single-cell HSE-SLR reporter assay (Supplementary Figure 3b), the continuous rise in the HSF1 transcriptional activity level for more than 12 h post UV exposure does not completely match with the Per2-Luc level, which begins to fall within 8 h post UV exposure. This difference suggests certain repressive mechanism to modulate *Per2* expression. p53-mediated transcriptional activity is reported to be increased by UV irradiation^21^. Additionally, recent studies have demonstrated that p53 function as a candidate mediator of circadian signaling through its suppression of *Per2* expression^22^. Hence, we investigated whether p53 regulates clock resetting by suppressing *Per2* expression upon UV stimulation. First, we examined the Per2-Luc response in wild-type and p53−/− MEFs^19^. In wild-type MEFs, Per2-Luc initially increased at 4–10 h post UV irradiation, followed by a decrease in luminescence. However, in p53−/− cells, the Per2-Luc surge was instead observed at 8–16 h, and the elevation was maintained for more than one day post irradiation, revealing that the longer-lasting Per2-Luc surge may be due to a lack of p53-mediated modulation of *Per2* expression during UV-triggered clock synchronization (Fig. 3a, b). p53 is known to inhibit *Per2* expression by blocking the binding of BMAL1 to the E-box sequence adjacent to the p53 response element (p53RE)^19^; therefore, p53 may suppress *Per2* expression after 4–10 h post UV irradiation in wild-type cells. Importantly, no evident circadian rhythm after UV irradiation was observed in p53−/− cells, whereas Dex treatment synchronized both cell types, indicating that the p53-mediated pathway is pivotal for UV-triggered clock synchronization (Fig. 3c).

Next, we conducted a ChIP analysis to determine whether p53 binds to the p53RE after UV irradiation (Fig. 3d). ChIP was performed at 2 h post irradiation to quantify the surge in p53 binding to respective elements and revealed that p53 binds to the p53RE adjacent to E-Box2, as well as to the p53RE in a known p53 target, namely, *Mdm2* gene, but not to the E-Box5 adjacent to the HSE2 in the *Per2* promoter (Fig. 2a). This observation supports the notion that p53 suppresses *Per2* expression by binding to p53RE during UV-triggered clock synchronization. Together, these results demonstrate that p53 is another critical transcription factor that modulates *Per2* expression during UV-triggered clock synchronization.

### HSF1 regulates p53 its their interaction during the reset

**Fig. 4:**
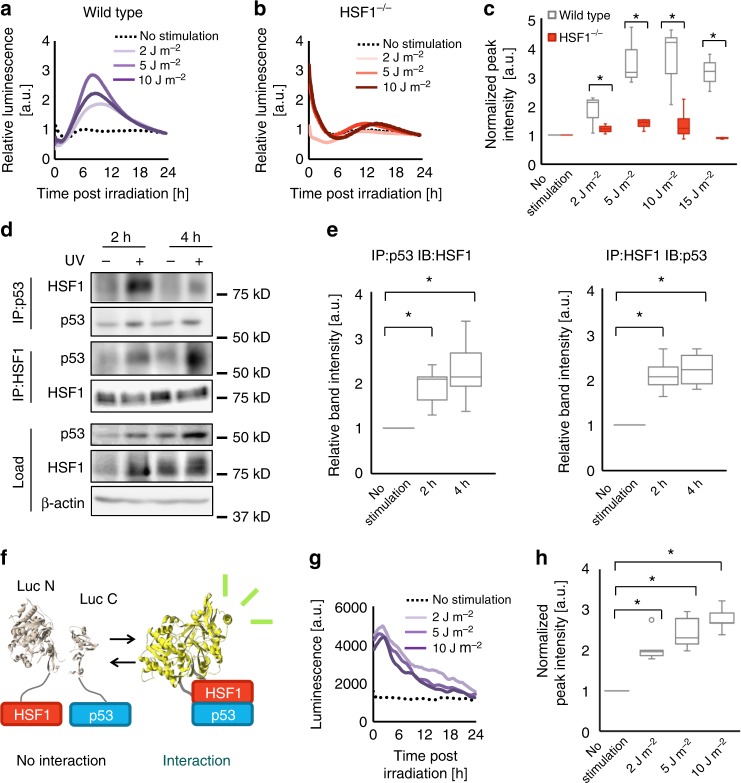
HSF1 interacts with p53 and governs p53 activation during UV-triggered clock synchronization. **a**–**c** Monitoring of p53 trans-activation with the p53RE-driven luciferase (p53RE-Luc) reporter. Dose-dependent induction of p53 activity after UV irradiation (254 nm) as monitored using NIH3T3 cells harboring the p53RE-Luc reporter (**a**). Dose-dependent induction of p53 activity after UV irradiation as monitored using HSF1−/− MEFs harboring the p53RE-Luc reporter (**b**). Comparison of dose-dependent p53RE activation between wild-type and HSF1−/− MEFs (**c**). *n* = 3. **d**, **e** Interaction between HSF1 and p53 as observed by coimmunoprecipitation. NIH3T3 cells were stimulated with UV irradiation (254 nm, 10 J m^−2^) and subjected to a coimmunoprecipitation assay at 2 or 4 h after stimulation. Representative blots of at least four independent experiments (**d**, *n* = 4 for the IP-HSF1 experiment and *n* = 5 for the IP-p53 experiment) are shown. See Supplementary Figure 14 for full-size blot images. Quantification of the band intensity of the coimmunoprecipitation assay (**e**). Values were normalized to the band intensity of actin in each corresponding sample and further normalized to the band intensity of the unirradiated sample. **f** A schematic illustrating the principle of the split-luciferase assay used to detect an interaction between HSF1 and p53. Upon their interaction, the two split fragments come into proximity with one another to reconstitute a full-length luciferase, thereby regaining a bioluminescent signal. **g** Split-luciferase complementation analysis after various strengths of UV irradiation. NIH3T3 cells harboring split-luciferase probes were irradiated with 2, 5, and 10 J m^−2^ UV irradiation. The temporal profiles of the split-luciferase reconstitution are shown. **h** Quantification of the maximum peak intensities relative to the non-irradiated control. *n* = 3. For all data, *p < 0.05 and ns not significant (*p* > 0.05), two-tailed *t*-test

Our results indicate that at least two transcription factors, namely, HSF1 and p53, are engaged in a UV-triggered clock synchronization mechanism. Therefore, we hypothesized that HSF1 and p53 cooperatively regulate *Per2* expression during clock synchronization to evoke protective responses against UV-induced cell stress. In support of this notion, associations between HSF1 and p53 were reported to affect the protective response against proteotoxic and genotoxic stimulations^13,23^. To test our hypothesis, we assessed p53-mediated transactivation by a reporter assay using a p53-response element-driven firefly luciferase (p53RE-Luc) after UV irradiation in wild-type and HSF1−/− MEFs. In wild-type cells, there was an increase in p53RE-Luc activity after UV irradiation at a dose within the circadian clock-resetting range. Together with the results from Fig. 3, these data suggest that an increase in p53-mediated transcriptional regulation is involved in UV-triggered clock synchronization (Fig. 4a). In contrast, HSF1−/− MEFs showed a substantially lower p53RE-Luc surge after UV irradiation than wild-type MEFs (Fig. 4b, c), demonstrating that HSF1 is pivotal for p53 activation after UV irradiation. We also evaluated p53 activation by quantifying the transgene product of p53 upon stimulation. Some transgene products, including *Bbc3* and *Cdkn1a*, showed lower expression in HSF1−/− than in wild-type cells, implying that HSF1 upregulates p53 during UV-triggered clock synchronization (Supplementary Figure 5). Conversely, p53−/− MEFs showed a similar UV-induced HSE-SLR surge to that of wild-type MEFs, suggesting that p53 does not affect the activation of HSF1 upon UV irradiation (Supplementary Figure 6a–d). These results show that HSF1 regulates p53 in a hierarchical manner, in which the presence of HSF1 is critical for p53 activation upon UV irradiation. To examine whether direct HSF1–p53-binding serves as the molecular basis for the HSF1–p53 interplay during UV-triggered clock synchronization, we conducted coimmunoprecipitation assays of HSF1 and p53 after UV stimulation (Fig. 4d and Supplementary Figure 6e). The immunoblot analysis for HSF1 in the p53-coimmunoprecipitates revealed an acute increase in p53-bound HSF1 after UV irradiation. Additionally, HSF1-immunoprecipitated p53 also increased, indicating direct interactions between HSF1 and p53 induced by UV irradiation. The temporal changes in the HSF1–p53 interaction were estimated by quantifying the intensities of the HSF1 and p53 immunostained bands after both coimmunoprecipitations, demonstrating increase in the HSF1–p53 interaction at 2–4 h post UV irradiation (Fig. 4e). To demonstrate the precise temporal kinetics of this interaction between HSF1 and p53 in living cells, a split-luciferase complementation assay was performed. In this assay, split fragments of a luciferase gene were fused to HSF1 and p53. With this technique, an interaction between HSF1 and p53 was observed due to the bioluminescence resulting from the reconstituted luciferase (Fig. 4f–h). The luminescence profile demonstrated that the HSF1–p53 interaction increased immediately after UV irradiation and began to decrease within 6 h post irradiation. These results suggest that a protein–protein interaction between HSF1 and p53 occurs after UV irradiation, and this interaction is presumably a cue that provokes the regulation of p53 activity by HSF1.

### BMAL1 regulates HSF1 and p53

**Fig. 5:**
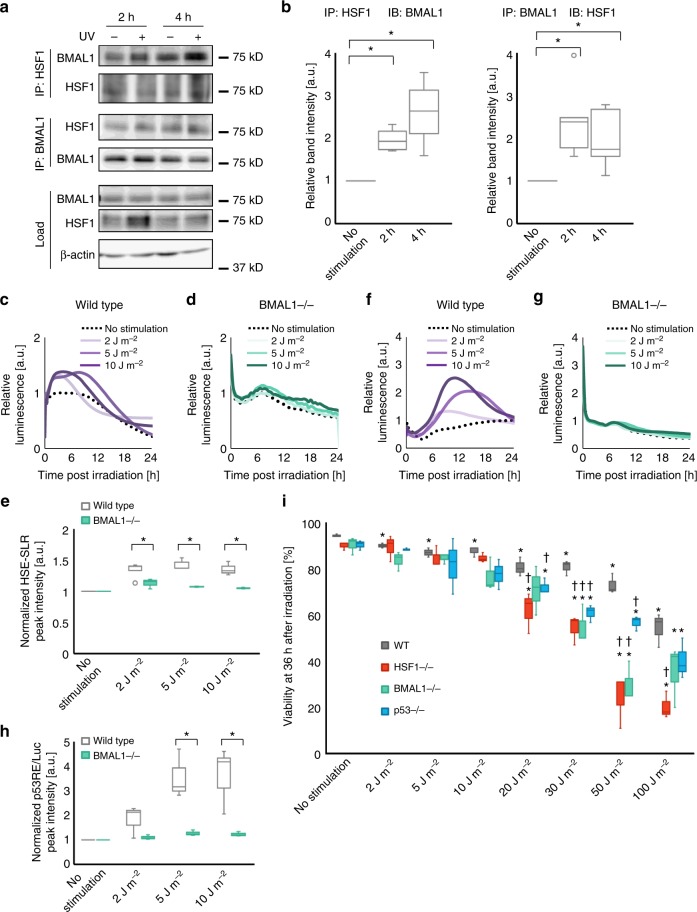
HSF1 interacts with BMAL1 during UV-triggered clock synchronization. **a**, **b** NIH3T3 cells were stimulated with UV irradiation (10 J m^−2^) and sampled at 2 or 4 h after stimulation. Samples were immunoprecipitated with either the anti-BMAL1 or anti-HSF1 antibody and immunoblotted with an antibody against the target protein indicated in the figure. Representative images of four independent experiments (**a**, *n* = 4 for the IP-HSF1 experiment and *n* = 5 for the IP-BMAL1 experiment) are shown. See Supplementary Figure 15 for full-size blot images. Quantification of the band intensity of the coimmunoprecipitation assay (**b**). Values were normalized to the band intensity of β-actin from each corresponding sample and further normalized to the band intensity of the non-irradiated sample. **c**–**e** Wild-type and BMAL1−/− MEFs harboring the HSE-SLR reporter were exposed to UV stimulation. The temporal profiles of the reporter luminescence signal for the representative sample of each stimulation condition for wild-type (**c**) and BMAL1−/− MEFs (**d**). Quantification of the peak intensity for each profile. Values were normalized to that of the unstimulated sample (**e**). *n* = 3. **f**–**h** Wild-type and BMAL1−/− MEFs harboring the p53RE-Luc reporter were exposed to UV stimulation. The temporal profiles of the reporter luminescence signal for the representative sample of each stimulation condition for wild-type (**f**) and BMAL1−/− MEFs (**g**). Quantification of the peak intensity for each profile (**h**). Values were normalized to that of the unstimulated sample. *n* = 3. **i** Cell viability of wild-type, BMAL1−/−, HSF1−/−, and p53−/− MEFs 36 h after different levels of UV exposure. Cell viability was calculated from trypan blue staining. *n* = 3. For all data. *p < 0.05 and ns: not significant (*p* > 0.05), from a two-tailed *t*-test except for **i**. For **i**, *p < 0.05, two-tailed *t*-test for the comparison between non-irradiated and irradiated samples; ^†^*p* < 0.05, two-tailed *t*-test for the comparison between wild-type MEFs and null mutants. Not significant (*p* > 0.05) unless indicated

Our previous study revealed that the interaction between HSF1 and the circadian transcription factor BMAL1 is critical for clock synchronization triggered by oxidative stress^6^. Therefore, we expected that the interplay between HSF1 and BMAL1 is also an important feature of the clock synchronization process after UV irradiation. Hence, we investigated the interplay between HSF1 and BMAL1 by coimmunoprecipitation (Fig. 5a and Supplementary Figure 6e). HSF1-bound BMAL1 increased at 2 and 4 h post UV irradiation. Consistent results were obtained from the blot for the BMAL1-bound fraction of HSF1, suggesting that HSF1 interacts with BMAL1 for at least 4 h during the UV-triggered clock synchronization (Fig. 5b). We then investigated whether the HSF1–BMAL1 interaction affects their transcriptional activity. Because arrhythmic Per2-Luc in HSF1−/− MEFs suggested impairments in the UV-triggered clock synchronization response caused by HSF1 deficiency (Fig. 1c), we hypothesized that the HSF1–BMAL1 interaction might mediate the initial surge of Per2-Luc upon UV exposure. To analyze the dependency of the HSR on BMAL1, we examined HSE-SLR profiles in BMAL1−/− MEFs. HSF1 activation upon UV stimulation was significantly impaired in BMAL1-deficient cells (Fig. 5c–e), indicating that crosstalk between BMAL1 and HSF1 is induced at the early stage post UV irradiation and that BMAL1 is indispensable for the UV-triggered activation of HSF1, as previously reported for the oxidative stress response^6^. Notably, unlike wild-type MEFs, BMAL1−/− MEFs did not exhibit apparent p53RE-Luc activation after UV irradiation (Fig. 5f–h). Because the p53 response was also inhibited in HSF1−/− MEFs, the impaired activation of p53RE-Luc in BMAL1−/− cells was likely a consequence of the lack of HSF1 activation due to BMAL1 deficiency.

The sequential interplay between BMAL1, HSF1, and p53 was demonstrated with split-luciferase assays by analyzing the precise temporal profiles of each interaction. In this experiment, we used U2OS cells, another model for circadian clock synchronization (Supplementary Figure 7a), because ectopic BMAL1 expression for the detectable split-luciferase assay was possible for U2OS cells but difficult for NIH3T3 cells. After UV irradiation, both the BMAL1–HSF1 and HSF1–p53 interactions were induced, as monitored with the split-luciferase assays (Supplementary Figure 7b and 7c). This result suggests that the BMAL1–HSF1 interaction precedes the HSF1–p53 interaction when comparing the peak times of the split-luciferase intensity (Supplementary Figure 7d). Moreover, cell viability assays of BMAL1, HSF1 and p53 null-mutant MEFs after different doses of UV irradiation revealed that all of the mutants showed dose-dependent decreases in ell viability (Fig. 5i). Cell viability was significantly decreased with the BMAL1 and HSF1 null-mutants, especially at higher doses of irradiation. This result demonstrates that BMAL1, HSF1, and p53 are all indispensable for cellular protection against UV irradiation, with the dominant role played by BMAL1 and HSF1 in cell survival. In summary, the circadian transcription factor BMAL1 likely coordinates the adaptive responses of HSF1 and p53 against UV irradiation to synchronize cellular circadian clocks.

### CT-dependent response of HSF1 and p53 to UV irradiation

**Fig. 6:**
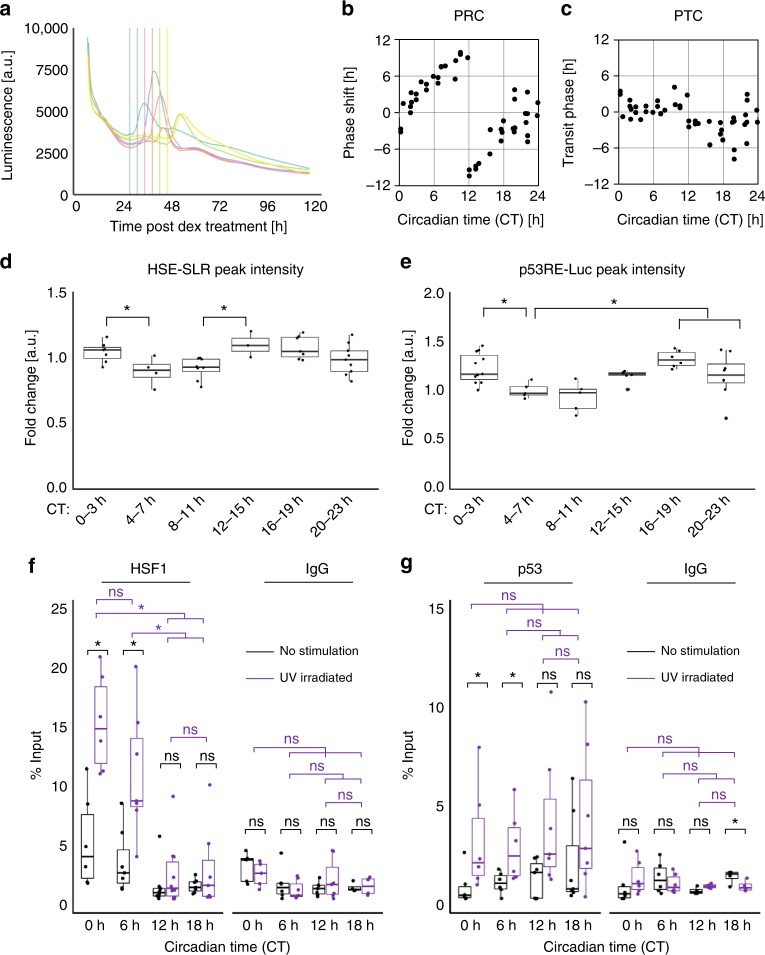
CT-dependent synchronization process upon UV irradiation. Analysis of circadian time (CT) dependency. NIH3T3 cells post synchronization by Dex (10 nM, 2 h) w ere irradiated with UV (10 J m^−2^) after 24–48 h of Dex treatment, defined as CT 0–24 h. For the analysis of the CT groups, values were tested by one-way ANOVA followed by a two-sample *t*-test. *p < 0.05, ns: not significant (*p* > 0.05) in two-tailed *t*-tests. **a** Representative Per2-Luc profiles for cells irradiated at 24, 28, 32, 36, 40, or 44 h with colors corresponding to the time when cells were irradiated, as shown in the vertical line with the same color. **b**, **c** The phase–response curve (**b**, PRC) and phase–transition curve (**c**, PTC) for the UV-triggered clock synchronization. Phase shifts and phase transitions were calculated using the first peak time post irradiation. **d** Time-dependent activation of HSF1 monitored by HSE-SLR. The CTs of the irradiation were divided into 6 groups: CTs 0–3 h, 4–7 h, 8–11 h, 12–15 h, 16–19 h, and 20–23 h. The fold change in the HSE-SLR peak intensity calculated by taking the ratio of the intensity between the peak and at the irradiated time for each CT group is shown. At least three replicates per group. ANOVA; *F* = 4.00, *p* = 6.0 × 10^−3^. **e** Time-dependent activation of p53 monitored by p53RE/E-box-Luc. The CTs of the irradiation were divided into six groups as in **d**. The fold change in the peak intensity calculated as in **d** for each CT group is shown. At least five replicates per group. ANOVA; *F* = 5.71, *p* = 6.0 × 10^−4^. **f**, **g** ChIP assay of HSE2 or p53RE on the *Per*2 promoter after UV irradiation. ChIP assay of HSE2 using anti-HSF1 antibody or mouse IgG as a negative control (**f**). ChIP assay of p53RE using anti-p53 antibody or mouse IgG as a negative control (**g**). There were at least six replicates for each group. Statistical values for ANOVA are listed in Supplementary Table 3

Because the above findings imply that the circadian trans-activator BMAL1 is likely an integrative regulator of UV-activated transcription factors HSF1 and p53, we hypothesized that UV irradiation triggers activation of these transcription factors in a CT-dependent manner. To test this hypothesis, we characterized the CT-dependent phase-shifting property of circadian clocks. NIH3T3 cells harboring Per2-Luc were synchronized with Dex and then subjected to UV irradiation for 24 to 48 h (defined as CT 0 to 24 h) post Dex treatment (Fig. 6a and Supplementary Figure 8a). By measuring the difference in the time of the peak expression of Per2-Luc in UV-irradiated and nonirradiated cells, we quantified the UV-induced phase-transition response of the clocks at each CT. The phase shifts over time were then plotted on a phase–response curve (PRC) to examine the CT dependency of UV-triggered clock synchronization (Fig. 6b). The PRC diagram shows that UV irradiation induced a phase shift in a CT-dependent manner. We also plotted the transition of the phase on a phase–transition curve (PTC) and found that UV-triggered synchronization of the clock by transitioning to a constant phase (CT = 0), indicating that UV stimulation is a type 0 resetting synchronization factor (Fig. 6c)^24^.

Because HSF1 and p53 have been shown to be cooperative mediators during UV-triggered clock synchronization, we further evaluated the CT dependency of the activation of these two stress-responsive transcription factors, as monitored with HSE-SLR and *Per2* promoter-originated p53RE-driven luciferase (p53RE/E-box-Luc) reporter assays, respectively. NIH3T3 cells expressing HSE-SLR or p53RE/E-box-Luc were treated with Dex and irradiated with UV at CT 0–24 h. The profiles before and after UV irradiation at the respective CTs were recorded for both reporters (Supplementary Figure 8b, c). From these profiles, the fold change in luminescence after UV irradiation was quantified (Fig. 6d, e). We observed CT-dependent activation patterns of transcription factors HSF1 and p53, as demonstrated by the difference in peak intensity values, which correspond to the degree of activation after irradiation. Notably, the profile of the first peak intensity of HSE-SLR post UV synchronization showed a significant CT-dependent pattern as evaluated by ANOVA, with a slightly higher intensity from CT 0–3 h compared to that from CT 4–7 h and from CT 12–15 h compared to that from CT 8–11 h. The highest intensity occurred between the CT 12–15 h and CT 16–19 h periods (Fig. 6d). The peak intensity of p53RE/E-box-Luc significantly fluctuated over the CT periods, exhibiting higher p53RE-Luc induction from CT 0–3 h, 16–19 h, and 20–23 h than from CT 4–11 h (Fig. 6e). Taken together, these results indicate the CT-dependent responses to UV stimulation of both HSF1 and p53 activity. Next, to examine the molecular basis for CT-dependent HSE-SLR and p53RE/E-box-Luc expression, we analyzed the CT-dependent binding of the transcription factors HSF1 and p53 to the *Per2* promoter after UV irradiation by ChIP. Dex-synchronized NIH3T3 cells were irradiated at the indicated CT and subjected to ChIP analysis (Fig. 6f, g). We found an evident CT-dependency for the amount of the HSF1 fraction that bound to DNA, where a significant amount of HSF1 was bound to DNA in response to UV irradiation at CT 0 h and CT 6 h. In contrast, a low level of HSF1 was bound to the *Per2* promoter after irradiation at CT 12 h and CT 18 h, suggesting that HSF1-mediated *Per2* regulation upon UV irradiation was absent or weak at these CTs. On the other hand, a significant and almost constant amount of p53 was bound to the *Per2* promoter in response to UV irradiation at all CTs, suggesting that p53-mediated *Per2* regulation was constitutively active throughout the day. These results demonstrate that HSF1 and p53 each bind to their respective consensus sequences on the *Per2* promoter in a CT-dependent manner to regulate *Per2* expression. CT-dependent HSF1 activation was also supported by transient inhibition of HSF1, where HSF1 activity was inhibited at the onset of UV irradiation by a reversible HSF1 inhibitor (KNK437, 100 µM), the effect of which was monitored using the Per2-Luc reporter (Supplementary Figure 9). We observed an impaired Per2-Luc surge with the addition of the HSF1 inhibitor at the onset of UV irradiation at CT 6 h, when HSF1 was predominantly bound to the *Per2* promoter after UV irradiation.

To examine the effect of HSF1 and p53 on the cellular protection network, we performed transcriptome data analysis of UV irradiated MEFs from NCBI database^25^. We found that together with the circadian clock system, a number of stress-responsive pathways including the apoptosis, cell cycle regulation and oxidative stress pathways, as well as the HSR and DNA damage response pathways, were activated by UV stimulation (Supplementary Figure 10). Notably, the expression of genes involved in the HSR pathway increased earlier (44% of upregulated genes responded within 3 h post irradiation) than the genes associated with the DNA damage response and cell cycle-related pathways (19 and 18%, respectively), supporting our findings that HSF1 modulates the expression of p53-related genes (Supplementary Figure 5). We then compared the differentially expressed genes for UV irradiation and our previous microarray data for ROS stimulation to determine whether similar protective pathways were regulated by these stressors (Supplementary Figure 11a and 11b). The results showed that the two forms of stimulation (UV, ROS) promoted the activation of similar pathways, but only some of the individual genes showing changes were regulated by both forms of stimulation, indicating that the mechanism for activating the cell protection system may differ between the two forms of stimulation. By mapping the transcriptome profile of gene expression after UV irradiation onto circadian and stress responsive pathway maps from WikiPathways^26,27^, we predicted the network for the cell protection response triggered by UV; this network comprised the activation of BMAL1–HSF1–p53 interplay and subsequent regulation of adaptive response such as DNA damage, anti-oxidation, cell cycle-related, and apoptosis pathways (Supplementary Figure 12).

**Fig. 7:**
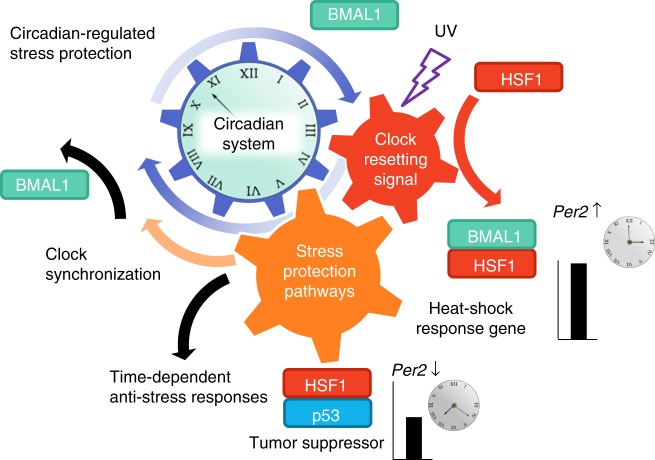
Resetting of the circadian clock synergistically activates adaptive response pathways in response to UV-induced cell stress. A hypothetical schematic diagram representing stress-triggered circadian synchronization mechanisms that synergistically activate adaptive responses. Stress triggers the clock-resetting signal mediated by BMAL1 through interactions between BMAL1, HSF1, and p53, which regulates *Per2* expression and synergistically activates the stress protective pathways, including the heat-shock response and DNA damage response represented by HSF1 and p53. Finally, these processes promote clock-resetting and time-dependent anti-stress responses

Taken together, these findings strongly suggest that CT-dependent HSF1 and p53 mediate the adaptive response to UV-induced stress and play critical roles during the synchronization of circadian clocks (Fig. 7).

## Discussion

In this study, based on our previously proposed notion that the stress-responsive clock synchronization process evokes protective responses via molecular networks among circadian clock and adaptive protection pathways, we investigated the interplay between the circadian timing system and stress response pathways during clock synchronization in response to UV irradiation^6,7^. In support of our notion, the central regulator for HSR pathway, HSF1, is identified as a circadian-recruited DNA-binding protein in the liver^9^, and changes in body temperature and heat-shock stimuli are now widely known to synchronize mammalian peripheral clocks via HSF1^7,28,29^. Several studies have demonstrated a correlation between molecular clocks and a tumor suppressor, p53. For example, p53 activity is controlled by BMAL1 in pancreatic cancer^30^, and p53 negatively regulates *Per2* expression^22^. Another group demonstrated that UV triggers time-dependent erythemal responses in human skin cells through CRY2 and p53^31,32^. These observations imply that the molecular clock responds to genotoxic stress by resetting its phases and simultaneously regulating stress response pathways. A CT-dependent phase shift of the clocks in response to critical cell stresses such as γ-ray irradiation has been reported^33,34^, indicating that the clock system is reset in response to such stress in a CT-dependent manner. Therefore, we hypothesized that stress responses occur in a CT-dependent manner, which likely originates from stress-responsive molecular processes during clock synchronization at each CT. Crucial to the pivotal molecular process, the BMAL1–HSF1–p53 interactions during and after UV-triggered clock synchronization were the focus of this study, and the CT-dependent context of these interactions was of particular interest.

To test our hypothesis, we first analyzed the clock-resetting responses after UV irradiation as manifested by the Per2-Luc surge after stimulation. According to a previous study, CT-dependent DNA repair is disrupted by BMAL1 knock-down, suggesting a protective role against UV^35^. Another study observed an increase in CRY1 and PER2 protein after γ-ray irradiation, and the *Per2* mutation reduced genotoxic-resistant gene expression, indicating that *Per2* may play a key role in DNA damage protection^11^. These observations support our data that indicate that *Per2* upregulation may be considered a marker for cellular protection against UV-induced stress. Using Per2-Luc as an indicator of the circadian rhythm, we have shown that HSF1 (Fig. 1c) and p53 (Fig. 3c) are necessary for UV-triggered clock synchronization. In support of this conclusion, the components of their corresponding pathways are upregulated after UV irradiation, probably via direct transactivation by HSF1 and p53 (Fig. 4a, b and Supplementary Figure 3b). Because the pivotal role of HSF1 is apparent in heat-shock-induced and ROS-induced clock synchronization^6,7^, HSF1 is considered to be a general mediator of cell stress-induced clock synchronization. p53 deficiency results in an impaired Per2-Luc surge after UV exposure, indicating that p53, along with BMAL1 and HSF1, may function to regulate clock synchronization. Because HSF1 is necessary for p53 trans-activation, this observation suggests that p53 mediates the clock-synchronizing signal in a partially HSF1-dependent manner (Fig. 4). Moreover, activation of both HSF1 and p53 are largely suppressed in BMAL1-lacking cells, demonstrating that BMAL1 is indispensable for the functions of HSF1 and p53 during UV-triggered clock synchronization (Fig. 5). Considering all of the above, we have elucidated a hierarchical network of transcription factors comprising the integrative regulator BMAL1, the BMAL1-regulated heat shock-responsive HSF1, and the HSF1 dependently activated p53. Our findings highlight the concept that stress-responsive cellular protection systems are activated while circadian clocks are reset.

Additionally, from the transcriptome data analysis we found some similar gene expression patterns between UV and ROS stimulation at the individual transcript level. Intriguingly, the expression of the circadian-related gene *Timeless* increased with both UV irradiation and ROS exposure (Supplementary Figure 11b). *Timeless* is known to be involved in circadian organization in response to DNA damage^36^, indicating that *Timeless* is another potential regulator of cellular stress adaptation. We also found increases in the expression of p53-related genes such as *Gadd45a* and *Trp53*; the HSR gene *Sod2*; and apoptosis/inflammatory-related genes such as *Akt1*, *Casp3,* and *Nfkbib*, implying that UV and ROS stress exposure may activate common adaptive pathways to the cell stresses via the expression of specific genes.

How cells, tissues and animals respond to daily environmental changes is an important question. Daily time-dependent molecular and physiological responses are coordinated by the circadian clock system. We found that both HSF1 and p53 were activated in a CT-dependent manner but with distinct patterns. Their peak times differed according to the CT, and their peak intensities showed oscillatory patterns with the peak at CT 16–19 h and the trough at CT 4–7 h (Fig. 6d, e). These results are indicative of the CT-dependent and differential actions of HSF1 and p53 in response to UV irradiation. As expected, we found daily fluctuations in the binding of HSF1 and p53 to the *Per2* promoter in CT-dependent ChIP assays, with predominant binding to the promoter occurring at CT 0 and CT 6 h (Fig. 6f, g).

HSF1 and p53 were CT dependently activated, and BMAL1 deficiency reduced their activity upon UV exposure; therefore, BMAL1 might control HSF1 and p53 activity during clock synchronization. Indeed, BMAL1 is considered to be a regulator of the p53 pathway through its control of the transcriptional activity of p53^16^. Moreover, the similar timing of the binding of HSF1 and p53 to the *Per2* promoter (Fig. 6f, g) and the reduced response of p53RE-Luc in HSF1-deficient cells (Fig. 4b) post UV exposure suggest that HSF1 and BMAL1 simultaneously control the transcriptional activation of p53 upon stimulation. These results indicate that the time-dependent binding of HSF1 and p53 to the *Per2* promoter is likely to be a major cause of the time-dependent response of Per2-Luc after UV irradiation.

The circadian clock is hypothesized to allow for escape from DNA replication in the cell cycle by predicting when DNA-damaging stimulation occurs^37^. More intensive analysis of the relationships between the circadian clock system and clock-timekeeping phenomena such as the cell division cycle and cellular metabolism is necessary. However, we believe that our model highlighting the interactive connections among the circadian clock system and stress response pathways during clock synchronization post stress exposure provides novel support for the concept that the circadian system activates stress-responsive cellular protection systems during the resetting of the circadian clock, controlling stress response pathways to vitally adapt to environmental changes.

## Methods

### Plasmid construction

A mouse *Per2* promoter-driven destabilized luciferase (Per2-Luc) reporter was developed as described previously^38^, and mouse Per2-Luc mutants were developed by site-directed mutagenesis. A reporter comprising the destabilized SLR, a red light emitting luciferase derived from the click beetle^39^, connected with 3 × HSE (HSE-SLR) was previously constructed^7^. Bioluminescence reporter expression vectors for HSE or the p53RE consensus sequence in the mouse *Per2* promoter were generated by PCR amplification and enzymatic digestion followed by ligation of the DNA fragments.

For the split-luciferase complementation assays, emerald luciferase (ELuc) cDNA (Toyobo) was split into two fragments, namely, the N-terminal fragment of firefly luciferase (N-Luc, 1-415 amino acid residues) and the C-terminal fragment of luciferase (C-Luc 393-542 amino acid residues). Full-length mouse p53 was ligated downstream of the C-terminal (ELucC) and inserted into the pcDNA3.1 vector (Invitrogen). Vectors containing the ELuc N-terminal fragment-fused mouse HSF1 were previously constructed^7^.

### Cell culture

Mouse fibroblast NIH3T3 cells (RIKEN cell bank, Japan), wild-type MEFs, BMAL1−/− MEFs^17^, HSF1−/− MEFs^18^, p53−/− MEFs^19^, human osteosarcoma U2OS cells (RIKEN cell bank, Japan), and plat-E cells (Cell Biolabs Inc.) were cultivated with Dulbecco’s modified eagle medium (D-MEM, Nacalai Tesque) supplemented with fetal bovine serum (FBS, Gibco) and 1% penicillin/streptomycin (Gibco). The morphology and behavior of the cell lines were consistent with their identities, but genetic validation was not carried out. Cell lines were tested for mycoplasma contamination by PCR and found to be negative.

Transfection of a probe DNA plasmid into cultured cells was performed using the TransIT-LT1 transfection reagent (Mirus bio) following the manufacturer’s protocol. For the generation of a stable cell line expressing the Per2-Luc or HSE-SLR reporter, retrovirus infection was conducted using a retrovirus produced in plat-E cells using polybrene (hexadimethrine bromide, final concentration of 0.24 mg/ml). Stable cell lines for the other reporter gene assays were generated by anti-biotic selection with hygromycin B after transfection of the probes.

### Real-time bioluminescence monitoring and data processing

Cells were synchronized by either Dex (10 nM, 2 h) or irradiation with UV light (254 nm cross-linker, UVP). As a model for genotoxic cell stimulation, a shorter UV wavelength (UV-C) was selected^40^. Before irradiation, the medium was replaced with phosphate-buffered saline (PBS) to avoid scattering and absorbance of UV-C light by the components of the medium. After UV-C irradiation, PBS was replaced to the original medium preserved from before irradiation. Real-time bioluminescence was monitored using Kronos Dio (ATTO, Japan) with acquisition intervals of 30 min for Per2-Luc and 10 min for other reporter experiments. For bioluminescence monitoring, the culture medium was supplemented with 0.1 mM d-luciferin (Wako, Japan).

The number indicated in each experiment refers to the number of samples analyzed with the same experimental setup. “Deviation from the moving average” in the *Y* axis indicates the raw values that were detrended (subtraction of a moving average) according to the program within the detector (Kronos Version 2.10.230; ATTO, Japan). The detrended values were further normalized by using maximum peak intensities over recorded values. To characterize the circadian rhythmicity, period and amplitude, we used Cosinor software downloaded from the Circadian Rhythm Laboratory Software home page (https://www.circadian.org/) to analyze each of the detrended and normalized luminescent profiles.

Real-time bioluminescence single-cell imaging was monitored under a BX-61 bioluminescence microscope (Olympus) using Metamorph Ver. 7.8.10.0 software. Single-cell tracking was performed using the TrackMate plugin in Fiji software (ImageJ 1.51u)^41^, according to the developer’s protocol. Values were normalized to maximum peak intensities over time.

### Immunoblot and immunoprecipitation assays

Cells cultivated in a culture dish were exposed to UV-C light (254 nm, 10 J m^−2^) and returned to the incubator until the predetermined time point for sample collection. At the sampling time point, cells were washed twice with ice-cold PBS and lysed with an NP-40 lysis buffer (10 mM Tris-HCl (pH = 7.4), 150 mM NaCl, 5 mM EDTA, 50 mM NaF and 0.5% NP-40) supplemented with a protease inhibitor cocktail (Complete, Roche) and a phosphatase inhibitor cocktail (PhosSTOP, Roche). The lysed samples were then centrifuged at 15,000 rpm for 20 min at 4 °C. For immunoprecipitation assays, the supernatant was collected, and a Triton X-100-containing lysis buffer was added to dilute the sample to 1 ml. The diluted sample was then incubated with protein G-sepharose beads overnight. The resultant products were separated and incubated with the anti-HSF1 antibody (1:1000, Cell Signaling Technology) or anti-p53 antibody (1:500, Santa Cruz Biotechnology) and protein G-sepharose beads for 6 h at 4 °C. The protein sepharose beads were collected and washed with PBS buffer three times and diluted with lysis buffer. The loading samples for SDS polyacrylamide gel electrophoresis (SDS-PAGE) were prepared by the addition of 0.2 equivalents of 5x sampling buffer (250 mM Tris-HCl (pH 7.6), 10% SDS, 25% glycerol, 5% 2-mercaptoethanol, 0.02% bromo phenol blue) to the supernatant of the centrifuged mixture. Immunoblotting was performed at 4 °C overnight using specific primary antibodies against the target protein, including primary antibodies against BMAL1 (80 ng/ml)^42^, HSF1 (1:1000, Cell Signaling Technologies #4356), Hsp70 (1:2000, Cell Signaling Technologies #4872), p53 (1:1000, Cell Signaling Technologies #9282) and actin (1:5000, Sigma AC-15 clone). Anti-rabbit IgG and anti-mouse IgG antibodies labeled with horseradish peroxidase (1:5000, GE Healthcare) were used as secondary antibodies. Chemiluminescence from the immunostained bands was detected with the ImageQuant LAS4000 Mini detection system (GE Healthcare), and the band intensities were quantified using Fiji software.

### Quantitative PCR

For quantification of circadian and heat-shock responsive genes, mRNA was collected from NIH3T3 cells exposed to UV-C light (10 J m^−2^) using Trizol reagent (Invitrogen). Collected mRNAs were reverse transcribed using PrimeScript Reverse Transcriptase (TakaraBio, Japan). The DNA fragments were amplified using THUNDERBIRD SYBR qPCR Mix (TOYOBO, Japan) following the manufacturer’s protocol. The fluorescence amplification curve was detected with a Thermal Cycler Dice Real Time System II with Software Ver. 5.11B (TakaraBio, Japan), and the quantification was performed from independent experiments. For quantification of the gene expression, values were normalized to the abundance at time 0. The primers used for the experiments are listed in Supplementary Table 1.

### Chromatin immunoprecipitation and qPCR

NIH3T3 cells cultivated in a culture dish were exposed to UV-C light (10 J m^−2^) and returned to the incubator until the predetermined time point for the sample collection. At the collection point, cells were washed with PBS (−) twice and fixed with 1% paraformaldehyde at room temperature for 5 min. The reaction was quenched with 1 M glycine and centrifuged to collect the cells. Cells were treated with RIPA buffer, incubated on ice for 20 min and subjected to sonication for at least 1 min for each sample. The supernatant was then collected, and a Triton X-100-containing lysis buffer was added to dilute the sample to 1 ml. The diluted sample was then incubated with protein G-sepharose beads overnight. The resultant products were separated and incubated with anti-HSF1 antibody, anti-p53 antibody or mouse IgG (Santa Cruz Biotechnology) with protein G-sepharose beads for 6 h at 4 °C. The protein sepharose beads were collected and washed with Triton X-100 buffer three times. Reverse crosslinking buffer (62.5 mM Tris-HCl pH 6.8, 200 mM NaCl, 2% SDS, and 10 mM DTT) was then added, and the samples were vortexed and incubated at 65 °C overnight. DNA fragments were collected by phenol-chloroform extraction followed by ethanol precipitation.

For quantification of transcription factor-bound DNA, qPCR targeting the mouse *Per2* promoter sequence using chromatin-immunoprecipitated samples was performed. The DNA fragments were amplified as described in the quantitative PCR (qPCR) section, and quantification was performed from independent ChIP experiments. The values represent the % input compared to the control, which was the total DNA subjected to the analysis, the value for which was set to 100%. The primers used for chromatin immunoprecipitation and qPCR experiments are listed in Supplementary Table 2.

### Transcriptome data analysis for UV and oxidative stress

Microarray data for UV-irradiated MEFs and hydrogen peroxide-treated NIH3T3 cells were downloaded from the Gene Expression Omnibus (GEO; http://www.ncbi.nlm.nih.gov/geo/) under the accession numbers GSE50930 and GSE47955^6,25^. Processed RMA values were used in further analysis. Gene expression levels were normalized to that of the unstimulated sample, and abundance changes were calculated as the log2 fold change. To ensure the reliability of the data, UV-regulated genes with inconsistent expression trends in the initial three time points (10, 30, and 60 min) were omitted from the analysis. Data were analyzed with custom-written MATLAB 2017a (MathWorks) code and R (ver. 3.4.3, https://www.R-project.org). The Venn diagram was generated using the “VennDiagram” package in R^43^, and pathway maps for the UV-stimulated gene expression profiles were generated using PathVisio 3.2.4 software^44,45^ with pathway information from WikiPathways^28,29^.

### Statistical analyses

All experiments were conducted with a specifically chosen sample size, and the sample number for each experiment is described in the figure legend. Error bars represent the standard deviation as indicated in the legends. For boxplots, a box is used to indicate the positions of the upper and lower quartiles; the interior of this box indicates the interquartile range, which is the area between the upper and lower quartiles. Whiskers are extended to the extrema of the distribution to 1.5 times of the interquartile range^46^. For the analysis of circadian fluctuation, one-way ANOVA followed by two-sided *t*-tests was performed. The samples with statistically significant p values by ANOVA are considered to have fluctuations. For the comparisons of values, two-sided *t*-tests were used. *p* values less than 0.05 (*p* < 0.05) were considered to be statistically significant. Statistical values are listed in Supplementary Table 3. All statistical tests were performed with R. Graphs were drawn either with Microsoft Excel 2016 or R.

### Code availability

All code is available from the authors upon request. MATLAB source code for drawing heatmap in Supplementary Figure 10 and Supplementary Figure 11 is provided as Supplementary Software 1.

## Electronic supplementary material

Supplementary Information

Supplementary Software 1

Description of Supplementary Software

## Data Availability

All relevant data are available from the authors upon request.
