# Supplementary material for: Cooperative interaction among BMAL1, HSF1, and p53 protects mammalian cells from UV stress
Source: Commun Biol. 2018 Nov 22;1:204. doi: 10.1038/s42003-018-0209-1 (PMC6250677; doi:10.1038/s42003-018-0209-1)
Supplement: Supplementary file 1 — Supplementary Information [file 42003_2018_209_MOESM1_ESM.pdf]

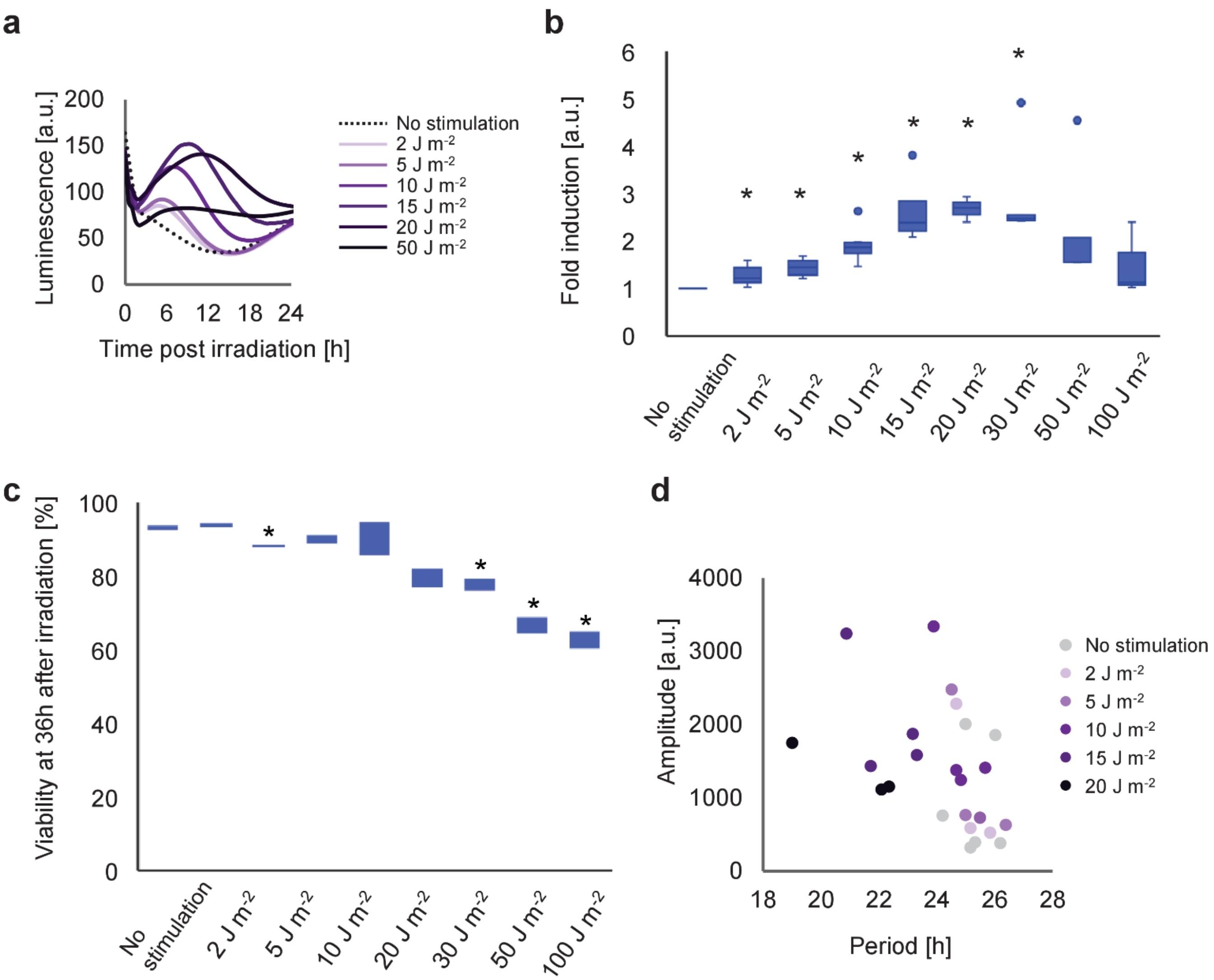

Supplementary Figure 1. UV triggers synchronization of the circadian clock.

a, **b** NIH3T3 cells harboring Per2-Luc were irradiated with different doses of UV light (254 nm), and the temporal luminescence profile after the irradiation was monitored. Representative Per2-Luc profiles of the initial responses of NIH3T3 cells after different doses of UV irradiation are shown (**a**). Relative Per2-Luc intensity change before and after UV irradiation (**b**). n = 3. *: $p<0.05$, two-tailed t-test for each group; not significant ($p>0.05$) unless mentioned. **c** Cell viability of NIH3T3 cells after 36 hours from UV exposure of different strengths calculated from trypan blue staining. n = 3, *: $p<0.05$, two-tailed t-test for each group; not significant ($p>0.05$) unless mentioned. **d** Scatter plot of period and amplitude of each profiles in Fig. 1a calculated using Cosinor program.

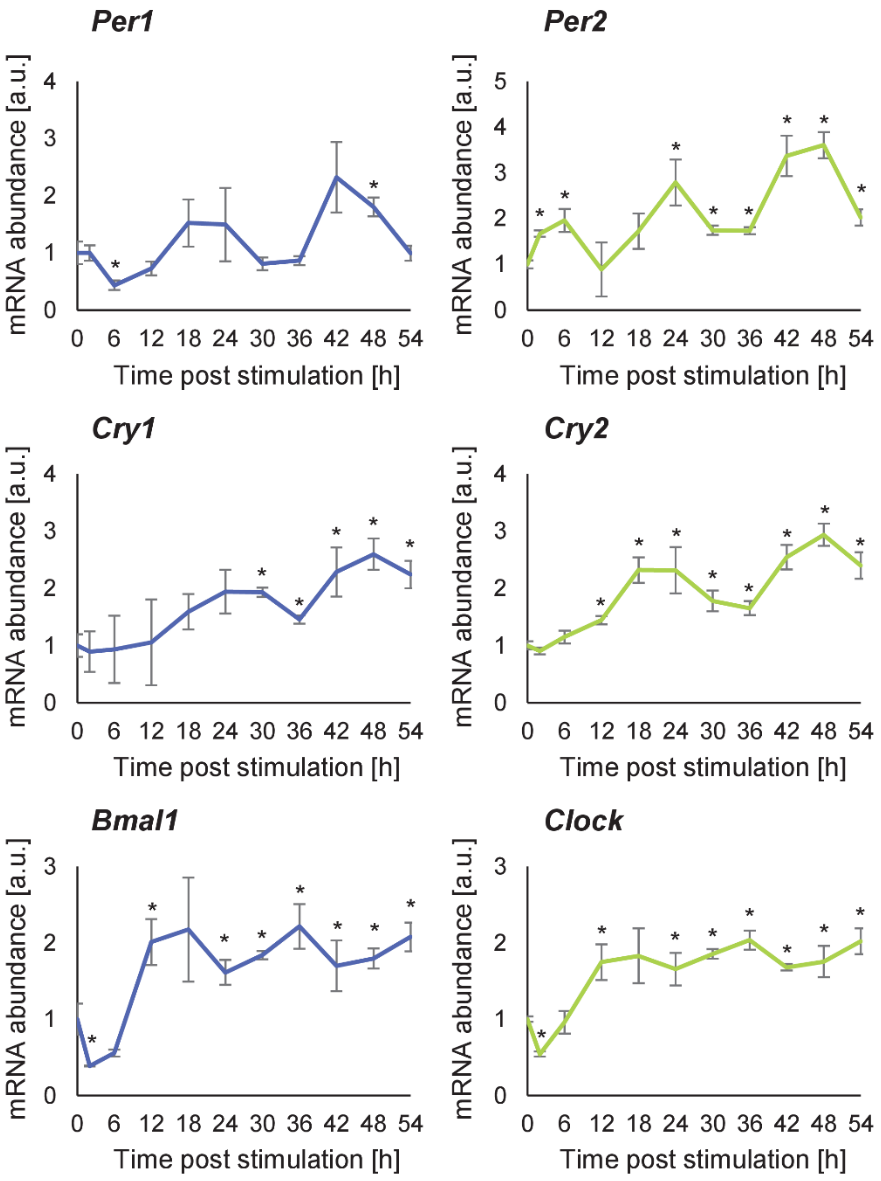

Supplementary Figure 2. Circadian profiles of core clock gene expression after UV irradiation.

mRNA levels of core clock genes after UV irradiation. NIH3T3 cells were irradiated with UV (10 J m^{-2}), and mRNA was collected at 0, 2, 6, 12, 18, 24, 30, 36, 42, 48 and 54 hours after the irradiation. mRNA levels of *BMAL1*, *CLOCK*, *Per1-3* and *Cry1-2* were quantified by qPCR. n = 3, error bar: SD, *: $p<0.05$, two-tailed t-test against abundance at time 0 for each group; not significant ($p>0.05$) unless mentioned.

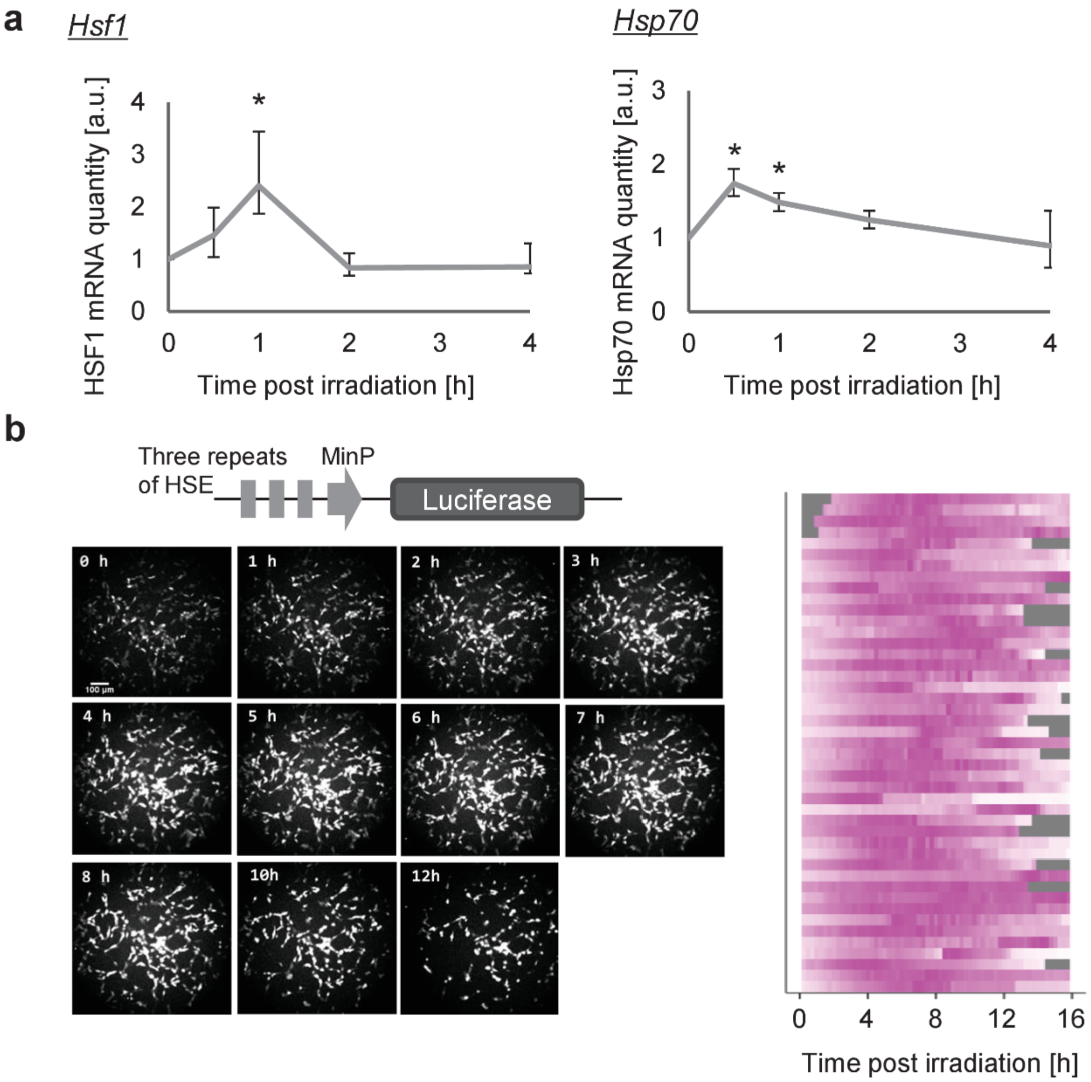

Supplementary Figure 3. HSF1 activation after UV irradiation.

a mRNA levels of HSR-related genes after UV irradiation. NIH3T3 cells were irradiated with UV (10 J m^{-2}), and mRNA was collected at 0, 0.5, 1, 2, and 4 hours after the irradiation. mRNA levels of HSF1 and Hsp70 were quantified by qPCR. n = 3, error bar: SD, *: $p<0.05$, two-tailed t-test for each group; not significant ($p>0.05$) unless mentioned. **b** NIH3T3 cells harboring HSE-SLR were irradiated with UV (10 J m^{-2}), and single-cell HSE-SLR profiles were monitored under a luminescence microscope. Images of representative time points are shown. Each row in the heat map shows the HSE-SLR profile of an individual cell that was tracked over time.

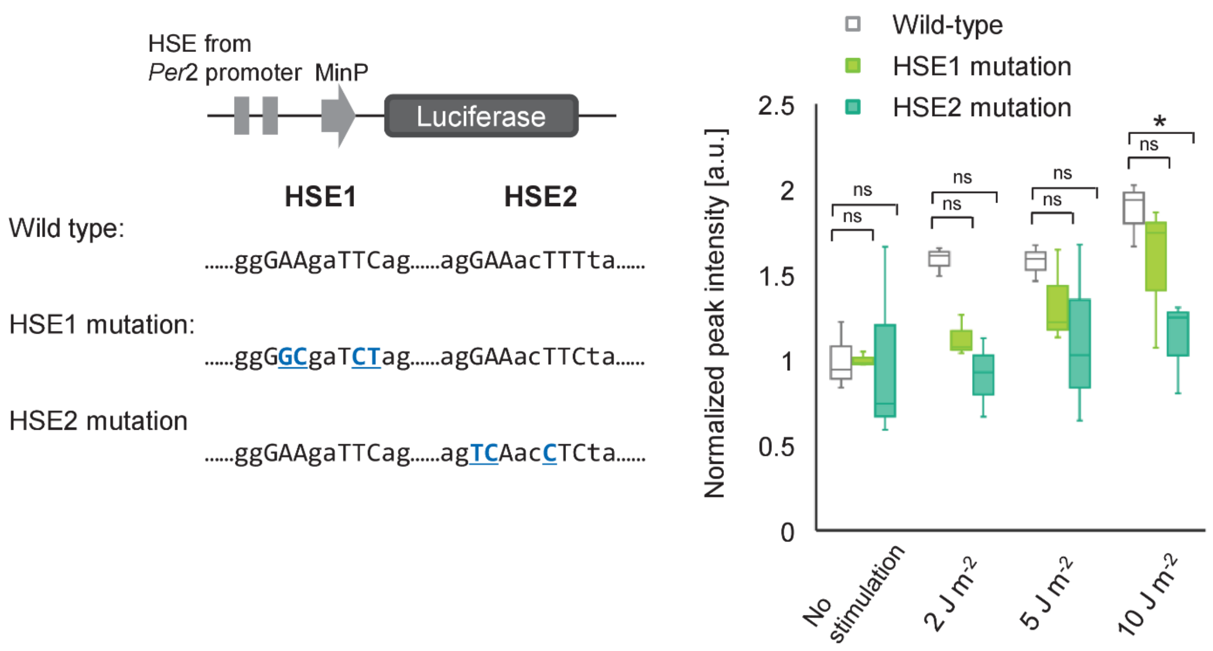

Supplementary Figure 4. HSE2 but not HSE1 of the *Per2* promoter is transcriptionally responsive after UV irradiation.

NIH3T3 cells were transfected with reporter vectors containing luciferase driven by HSE sequences of the mouse *Per2* promoter. Each HSE site was mutated to a non-HSE consensus sequence to make mutated HSE reporters. The HSE1 mutation probe corresponds to a reporter with mutated HSE1, and the HSE2 mutation probe corresponds to a reporter with mutated HSE2. Peak intensity after UV irradiation was quantified. n = 3 *: $p<0.05$, ns: not significant ($p>0.05$), two-tailed t-test.

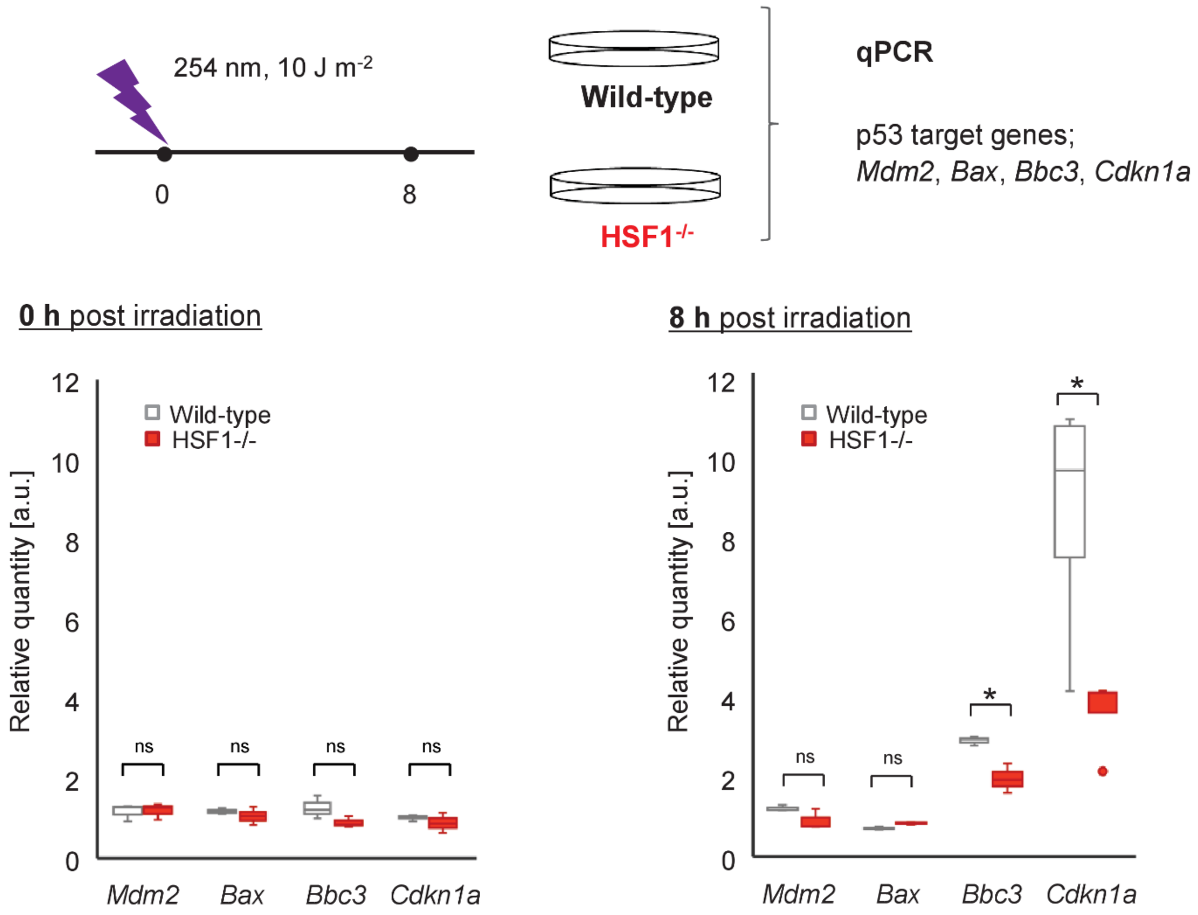

Supplementary Figure 5. p53 target gene expression is repressed in HSF1-/- cells after UV irradiation. The expression levels of p53 target genes, *Mdm2*, *Bax*, *Bbc3* and *Cdkn1a*, in wild-type and HSF1-/- cells were measured by qPCR. Cells were irradiated with UV (10 J m^{-2}) and subjected to qPCR at 0 and 8 h post stimulation. n = 3, *: p<0.05, ns: not significant (p>0.05), two-tailed t-test.

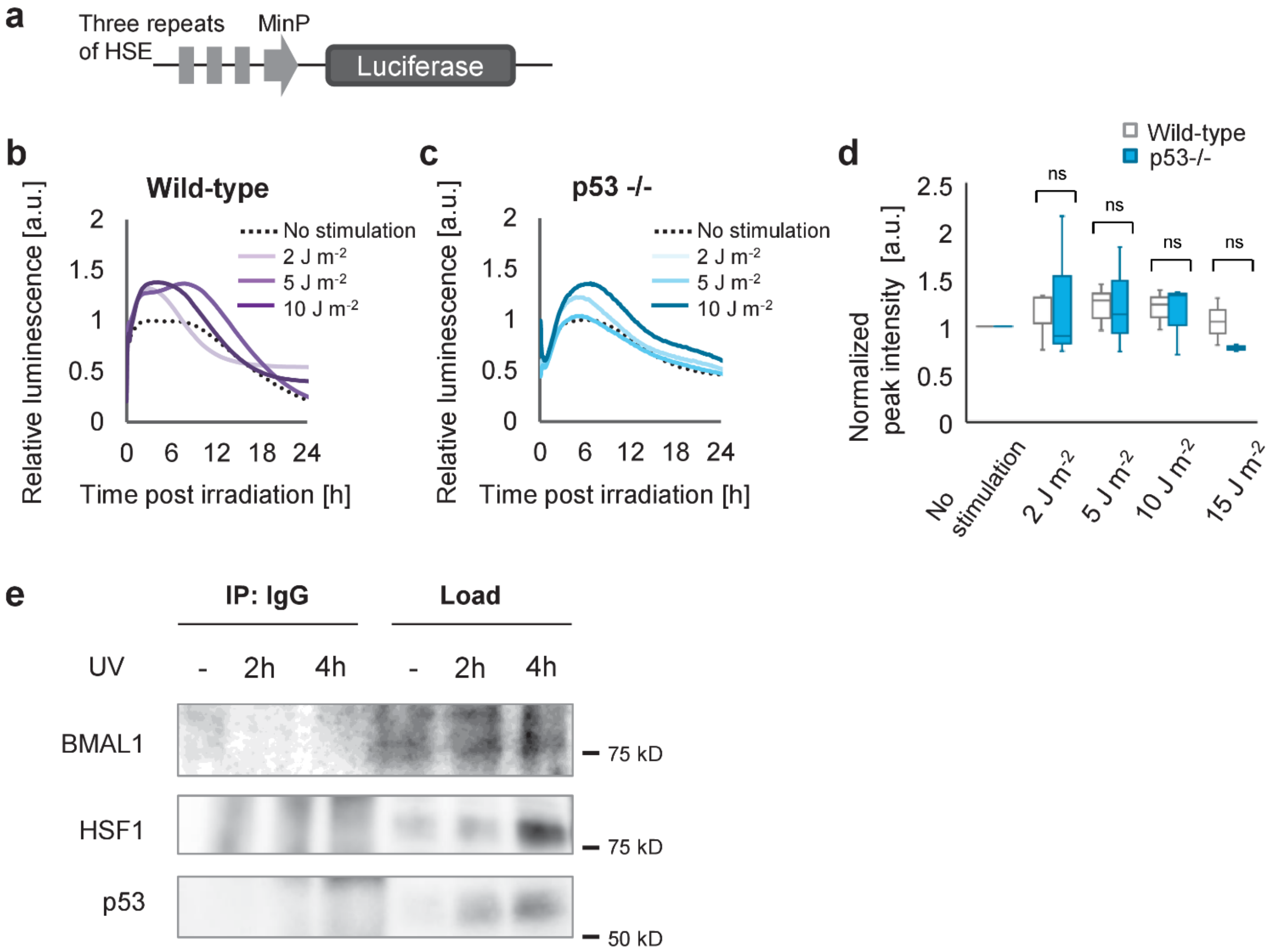

Supplementary Figure 6. p53 deficiency does not affect UV-triggered activation of HSF1.

a-d Dose-dependent induction of HSF1 activity after UV irradiation was monitored by MEF cells harboring the HSE-SLR reporter. Schematic diagram of HSE-SLR reporter (**a**). Dose-dependent induction of HSF1 activity after UV irradiation monitored by wild-type (**b**) and p53-/- MEFs (**c**) harboring the HSE-SLR reporter. Comparison of the dose-dependent p53RE activation between wild-type and p53-/- MEFs (**d**). Luminescence intensity at 0 h and 8 h (the first peak) post UV irradiation was used for the quantification. n = 3, ns: not significant ($p>0.05$), two-tailed t-test. **e** The negative control for immunoprecipitation assays using mouse-IgG, corresponding to Fig. 4d and 5a. NIH3T3 cells were stimulated with UV irradiation (254 nm, 10 J m^{-2}) and immunoprecipitated with mouse-IgG at 2 or 4 hours after stimulation. Representative blots for immunoblotting assay against BMAL1, HSF1 and p53 are shown. See Supplementary Figure 16 for full-size blot images.

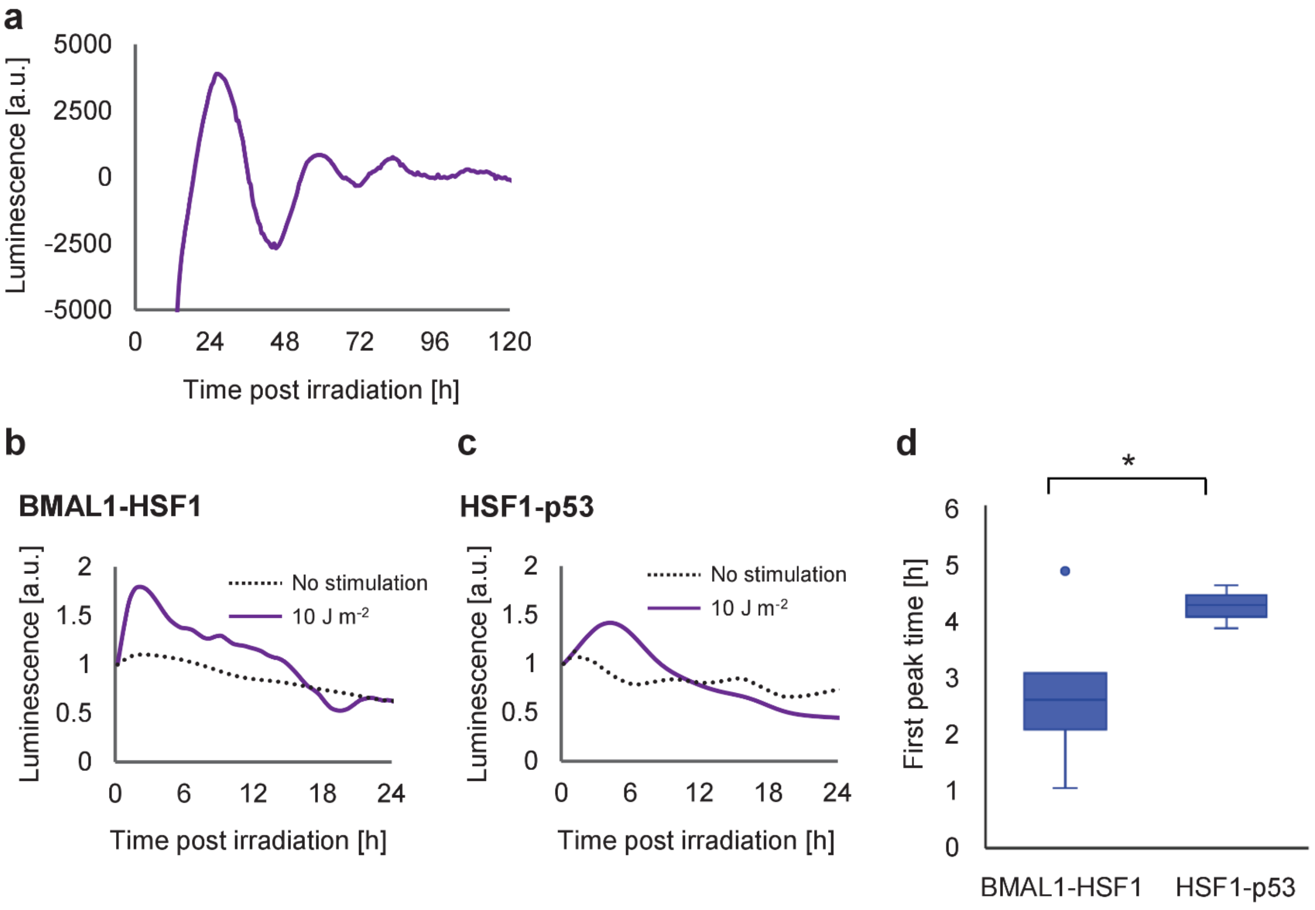

Supplementary Figure 7. Hierarchal regulation of BMAL1–HSF1–p53.

a Synchronization of Per2-Luc after UV irradiation (10 J m^{-2}) in U2OS cells. Raw values were detrended to emphasize circadian oscillation. **b-d** Split-luciferase complementation assay to monitor BMAL1–HSF1 and HSF1–p53 interactions. Averaged luminescence profiles of reconstituted luciferase for BMAL1–HSF1 (**b**) or HSF1–p53 (**c**) interactions after UV irradiation. Quantification of first peak time after the irradiation (**d**). n = 3, *: $p<0.05$, two-tailed t-test.

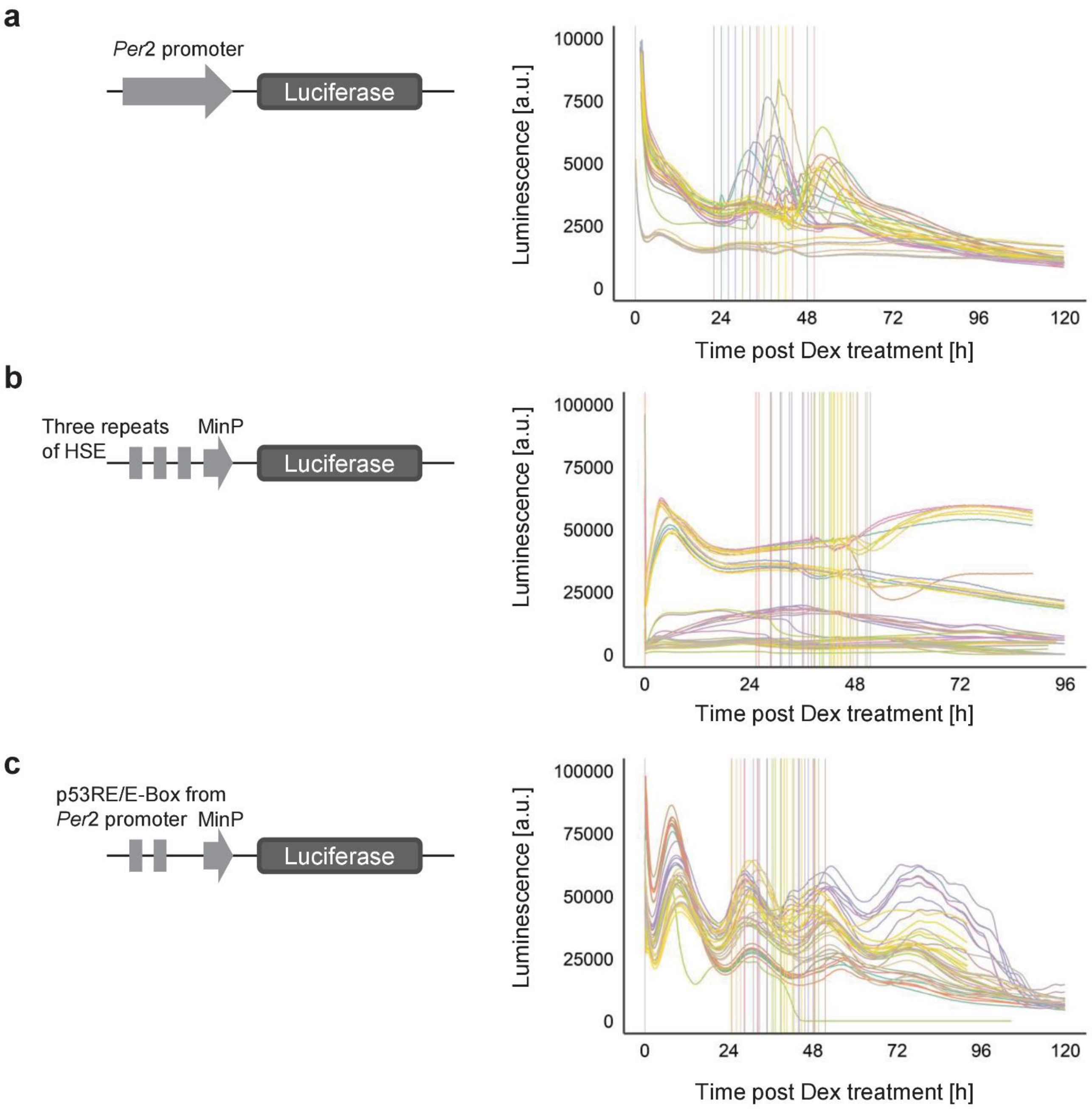

Supplementary Figure 8. (Corresponds to Fig. 6) Reporter profiles with UV irradiation at various CTs. **a** All measured Per2-Luc profiles upon UV irradiation at various CTs used for plotting the PRC and the PTC (Corresponds to Fig. 6a-c). Dex-synchronized NIH3T3 cells were irradiated with UV (10 J m^{-2}) at 24-48 hours (CT 0-24 h) post Dex treatment. The color of each vertical line corresponds to the time at which cells were irradiated; thereafter, each Per2-Luc profile, each a corresponding color, is shown. **b** All measured HSE-SLR profiles upon UV irradiation at various CTs used for the calculation of the first peak time and their peak intensity in Fig. 6d. Dex-synchronized NIH3T3 cells were irradiated with UV (10 J m^{-2}) at 24-48 h (CT 0-24 h) post Dex treatment. The color of each vertical line corresponds to the time at which cells were irradiated; thereafter, each HSE-SLR profile, each a corresponding color, is shown. **c** All measured p53RE/E-box-Luc profiles upon UV

irradiation used for the calculation of the first peak time and their peak intensity in Fig. 6e. Dex-synchronized NIH3T3 cells were irradiated with UV (10 J m^{-2}) at 24-48 h (CT 0-24 h) post Dex treatment. The color of each vertical line corresponds to the time at which cells were irradiated; thereafter, each p53RE/E-box-Luc profile, each a corresponding color, is shown.

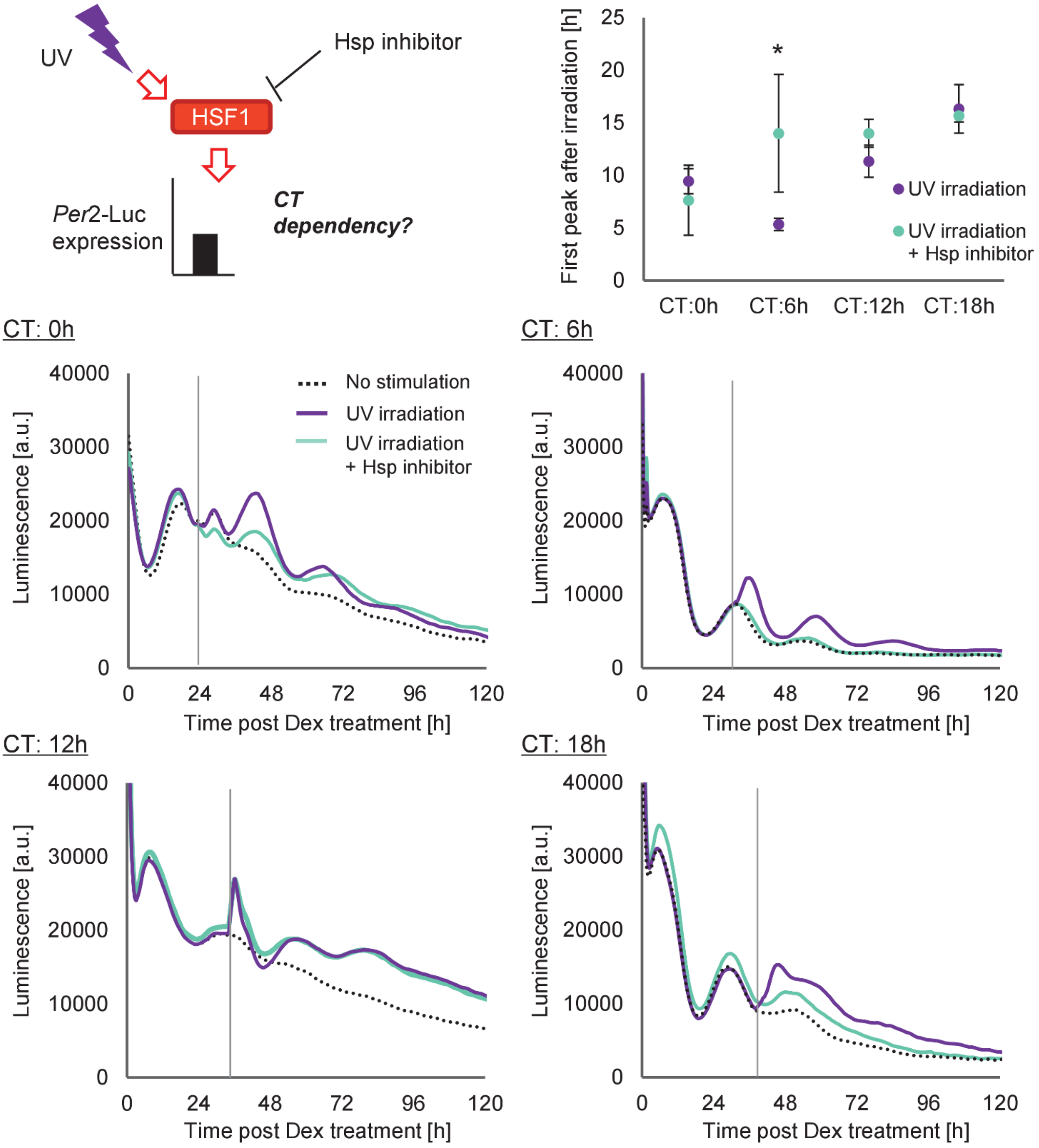

Supplementary Figure 9. Reversible inhibition of HSF1 at the onset of UV irradiation impairs the Per2-Luc surge in a time-dependent manner.

Per2-Luc profiles upon UV irradiation at various CTs with or without transient HSF1 inhibition by 100 µM KNK437. Dex-synchronized NIH3T3 cells were irradiated with UV (10 J m^{-2}) at 24-48 h (CT 0-24 h) post Dex treatment. KNK437 was added to the medium 15 min before the stimulation, and the medium was replaced with fresh medium 10 min after the irradiation. The first peak time after the irradiation was calculated, and the effect of KNK437 was compared to that of the untreated sample. Error bar: SD, n = 3, two-tailed t-test, *: p<0.05; not significant (p>0.05) unless mentioned.

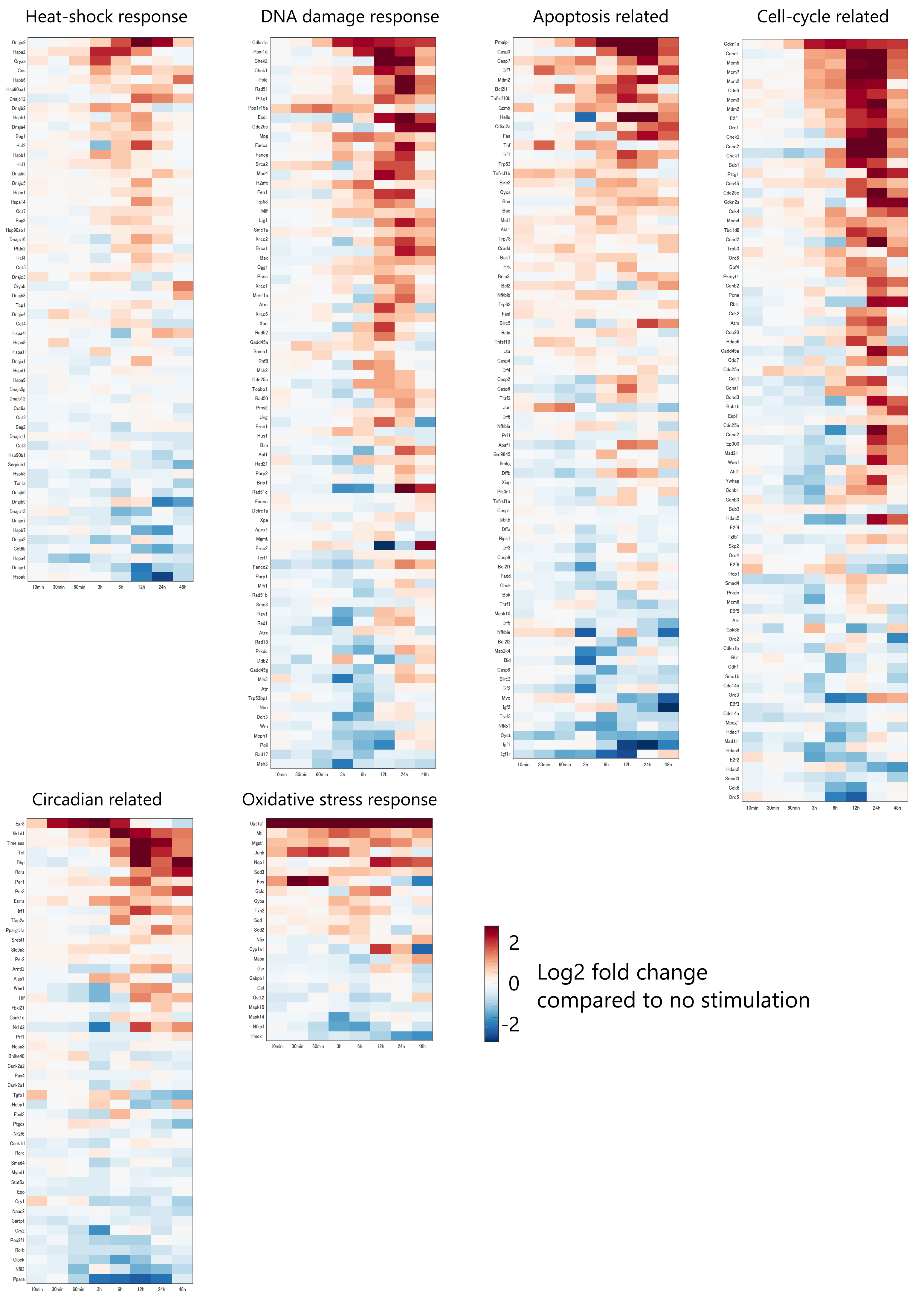

Heat-shock response
DNA damage response
Apoptosis related
Cell-cycle related
Circadian related
Oxidative stress response
10min
30min
60min
3h
6h
12h
24h
48h
2
0
-2
Log2 fold change
compared to no stimulation

Supplementary Figure 10. Expression profiles of genes related to the clock and stress response pathways affected by UV irradiation.

Gene expression profiles of UV (20 J m^{-2})-treated MEF cells reanalyzed from datasets from the microarray database. Values corresponding to the log2 fold change compared to those of the non-stimulated sample are color-coded from red, indicating increased expression, to blue, indicating decreased expression after UV stimulation. Gene expression profiles of apoptosis-related, cell-cycle-related, circadian-related, HSR, oxidative stress response and DNA damage response pathways extracted from the dataset are shown.

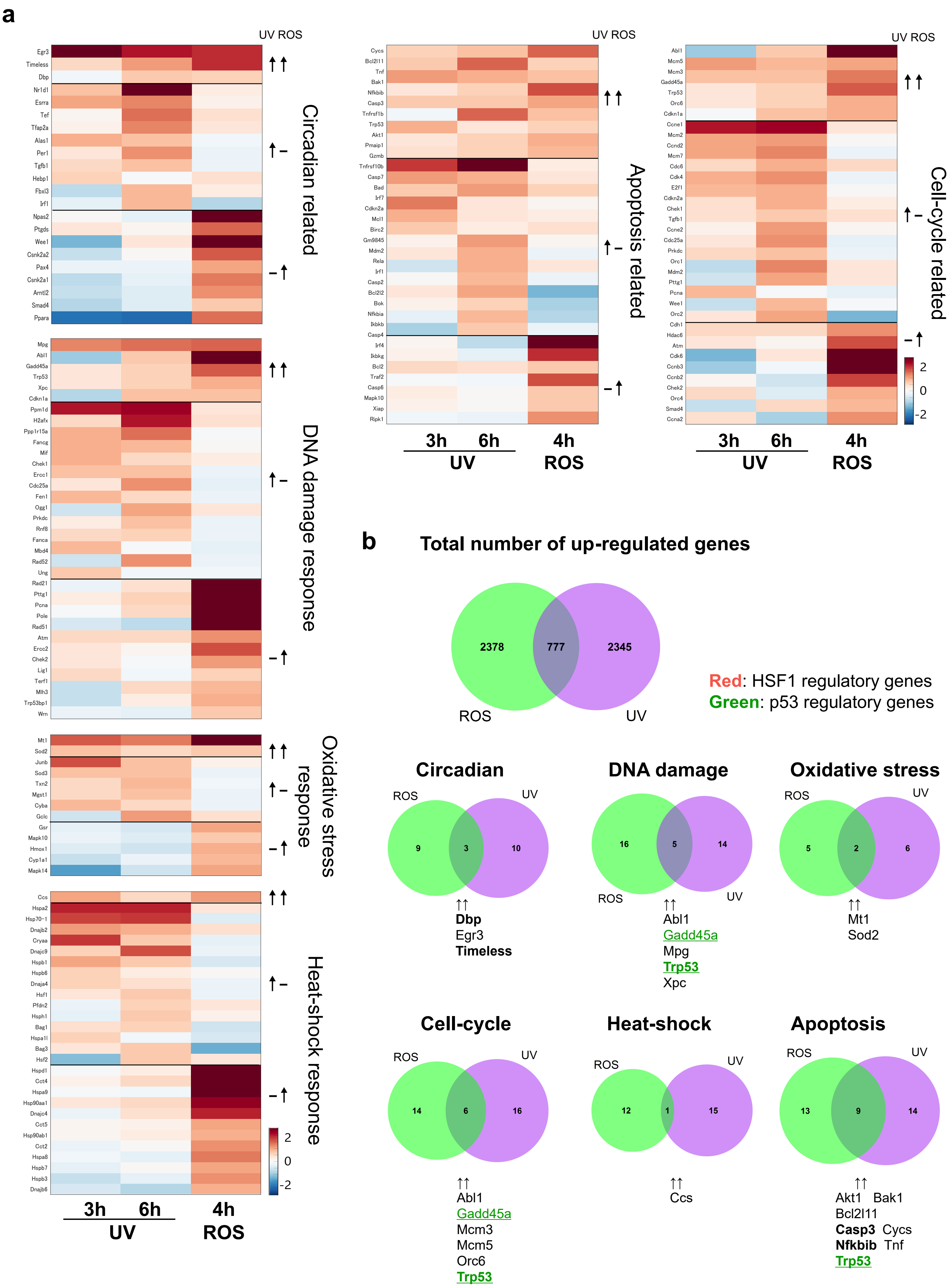
a
UV ROS
Circadian related
DNA damage response
Oxidative stress response
Heat-shock response
Apoptosis related
Cell-cycle related
2
0
-2
3h
6h
UV
4h
ROS
b
Total number of up-regulated genes
2378
777
2345
ROS
UV
Red: HSF1 regulatory genes
Green: p53 regulatory genes
Circadian
ROS
UV
9
3
10
Dbp
Egr3
Timeless
DNA damage
16
5
14
ROS
UV
Abl1
Gadd45a
Mpg
Trp53
Xpc
Oxidative stress
ROS
UV
5
2
6
Mt1
Sod2
Cell-cycle
ROS
UV
14
6
16
Abl1
Gadd45a
Mcm3
Mcm5
Orc6
Trp53
Heat-shock
ROS
UV
12
1
15
Ccs
Apoptosis
ROS
UV
13
9
14
Akt1 Bak1
Bcl2l11
Casp3 Cycs
Nfkbib Tnf
Trp53

Supplementary Figure 11. Similarly, or differentially regulated clock and stress-responsive genes after UV and oxidative stress exposure-induced clock synchronization.

Comparison of the gene expression profiles of UV (20 J m^{-2})-treated MEFs as in Supplementary Figure 10 and hydrogen peroxide-treated (ROS; 5 mM, 10 min) NIH3T3 cells. Genes with a fold change of at least 1.5 were defined as "responsive genes" for each condition. **a** Circadian and several stress response-related transcripts were extracted and depicted as a heat map of the log2 fold change compared to that of the non-stimulated sample. Expression profiles at 3 h or 6 h post UV stimulation and 4 h post ROS stimulation are shown. Among these responsive genes, genes whose expression increased in a similar pattern post UV and ROS stimulations, those whose expression increased only in response to UV stimulation, and those whose expression increased only in response to ROS stimulations were clustered respectively (up arrow indicates upregulated genes). **b** Venn diagram showing the number of responsive genes that were increased similarly or differentially in each stimulation condition. The number in each area shows the number of genes associated with each category. Gene names for transcripts that were upregulated by both stimulations (at least 1.5-fold change at either 3 h or 6 h post UV stimulation and at 4 h post ROS stimulation) are shown below the Venn diagram. Genes that are considered to be important for this study are indicated in bold characters. Red and green colored gene names indicate HSF1 regulatory or p53 regulatory genes, respectively.

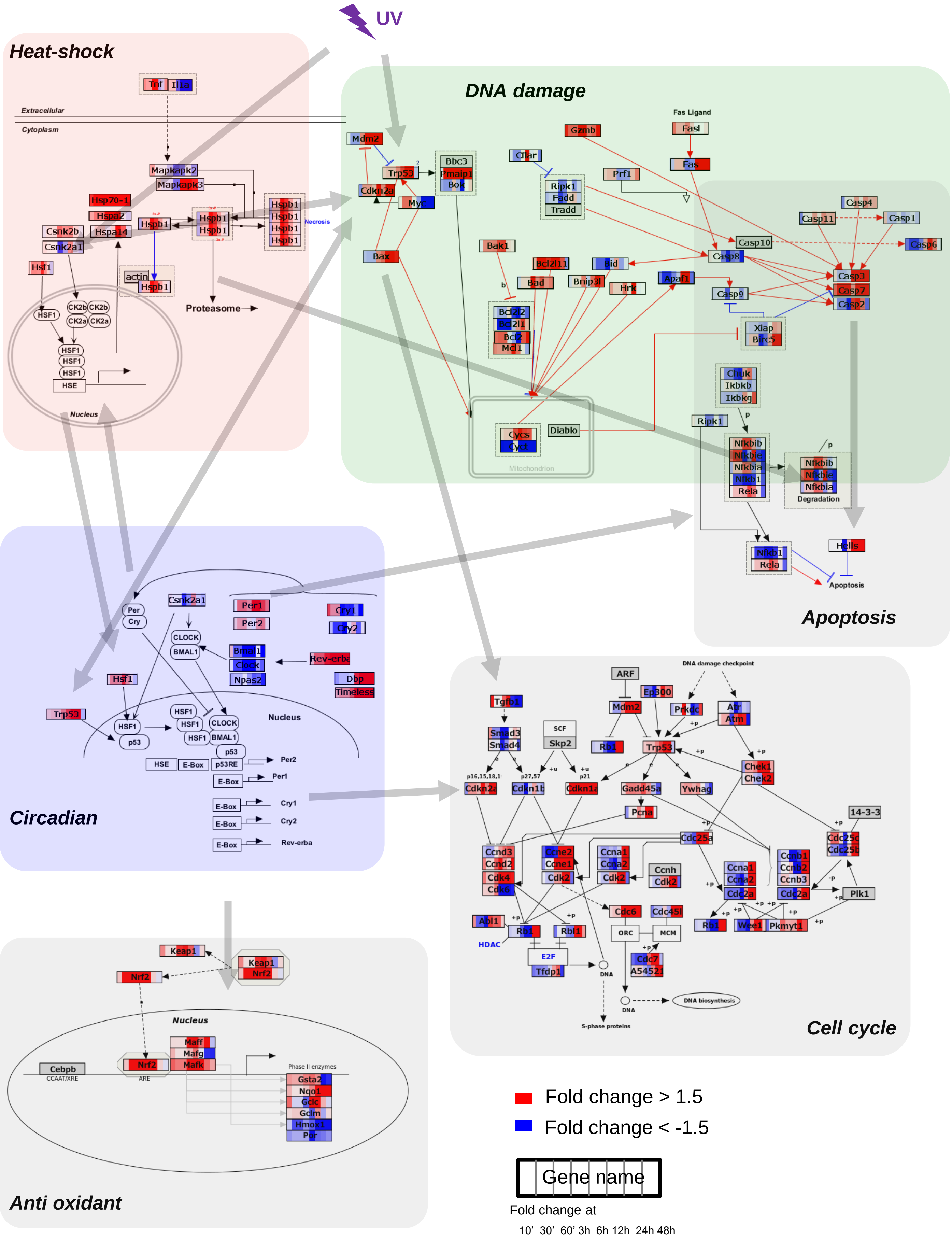
UV
Heat-shock
Extracellular
Cytoplasm
Tnf
Il1a
Mapkapk2
Mapkapk3
Hsp70-1
Hspa2
Hspa14
Csnk2b
Csnk2a1
Hsf1
Hspb1
actin
Proteasome
Necrosis
HSF1
CK2b
CK2a
HSE
Nucleus
DNA damage
Fas Ligand
Fasl
Fas
Gzmb
Mdm2
Trp53
Cdkn2a
Myc
Bbc3
Pmaip1
Bok
Cflar
Ripk1
Fadd
Tradd
Prf1
Bax
Bak1
Bcl2l11
Bid
Bad
Bnip3l
Hrk
Apaf1
Bcl2l2
Bcl2l1
Bcl2
Mcl1
Cycs
Cyct
Diablo
Mitochondrion
Casp4
Casp11
Casp1
Casp10
Casp8
Casp6
Casp3
Casp7
Casp9
Casp2
Xiap
Birc5
Chuk
Ikbkb
Ikbkg
Ripk1
Nfkbib
Nfkbie
Nfkbia
Nfkb1
Rela
Degradation
Hells
Apoptosis
Circadian
Per
Cry
Csnk2a1
CLOCK
BMAL1
Per1
Per2
Cry1
Cry2
Bmal1
Clock
Npas2
Rev-erba
Dbp
Timeless
Hsf1
Trp53
p53
p53RE
E-Box
Rev-erba
Cell cycle
DNA damage checkpoint
ARF
Ep300
Prkdc
Atr
Atm
Tgfb1
Smad3
Smad4
SCF
Skp2
Rb1
Chek1
Chek2
Cdkn2a
Cdkn1b
Cdkn1a
Gadd45a
Ywhag
Pcna
14-3-3
Cdc25a
Cdc25c
Cdc25b
Ccnd3
Ccnd2
Cdk4
Cdk6
Ccne2
Ccne1
Cdk2
Ccna1
Ccna2
Ccnh
Cdk7
Ccnb1
Ccnb2
Ccnb3
Cdc2a
Plk1
Abl1
Rbl1
Cdc6
Cdc45l
ORC
MCM
Wee1
Pkmyt1
HDAC
E2F
Tfdp1
Cdc7
A54521
DNA
DNA biosynthesis
S-phase proteins
Anti oxidant
Keap1
Nrf2
Maff
Mafg
Mafk
Cebpb
CCAAT/XRE
ARE
Phase II enzymes
Gsta2
Nqo1
Gclc
Gclm
Hmox1
Por
Fold change > 1.5
Fold change < -1.5
Gene name
Fold change at
10' 30' 60' 3h 6h 12h 24h 48h

Supplementary Figure 12. UV-induced signaling pathways and their respective expression profiles.
Pathway maps representing UV stress-responsive expression genes for circadian, HSR, DNA damage-related, apoptosis-related, cell-cycle-related and anti-oxidative pathways. Pathway maps were generated using PathVisio software with maps adopted from WikiPathways. Arrow indicates signaling pathways hypothesized to be activated upon UV irradiation. Gene names are color-coded with the time course fold change after UV irradiation. Arrows indicate mutual control among these pathways.

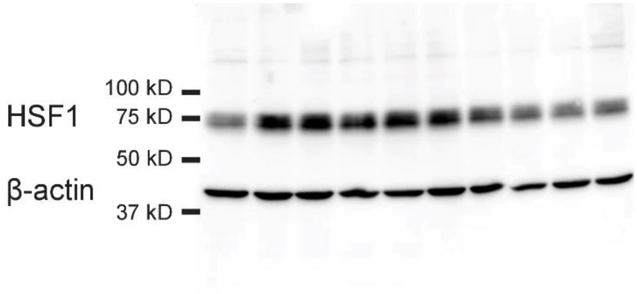

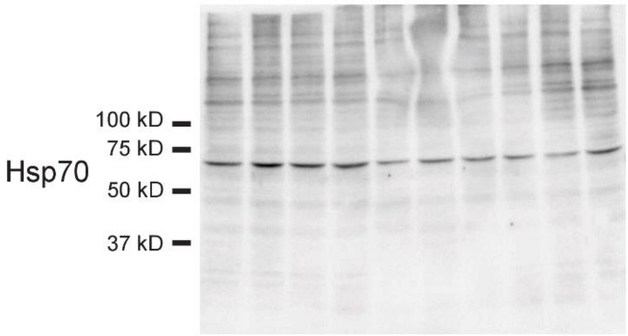

Supplementary Figure 13.

Full sized immunoblots corresponding to Fig. 1d.

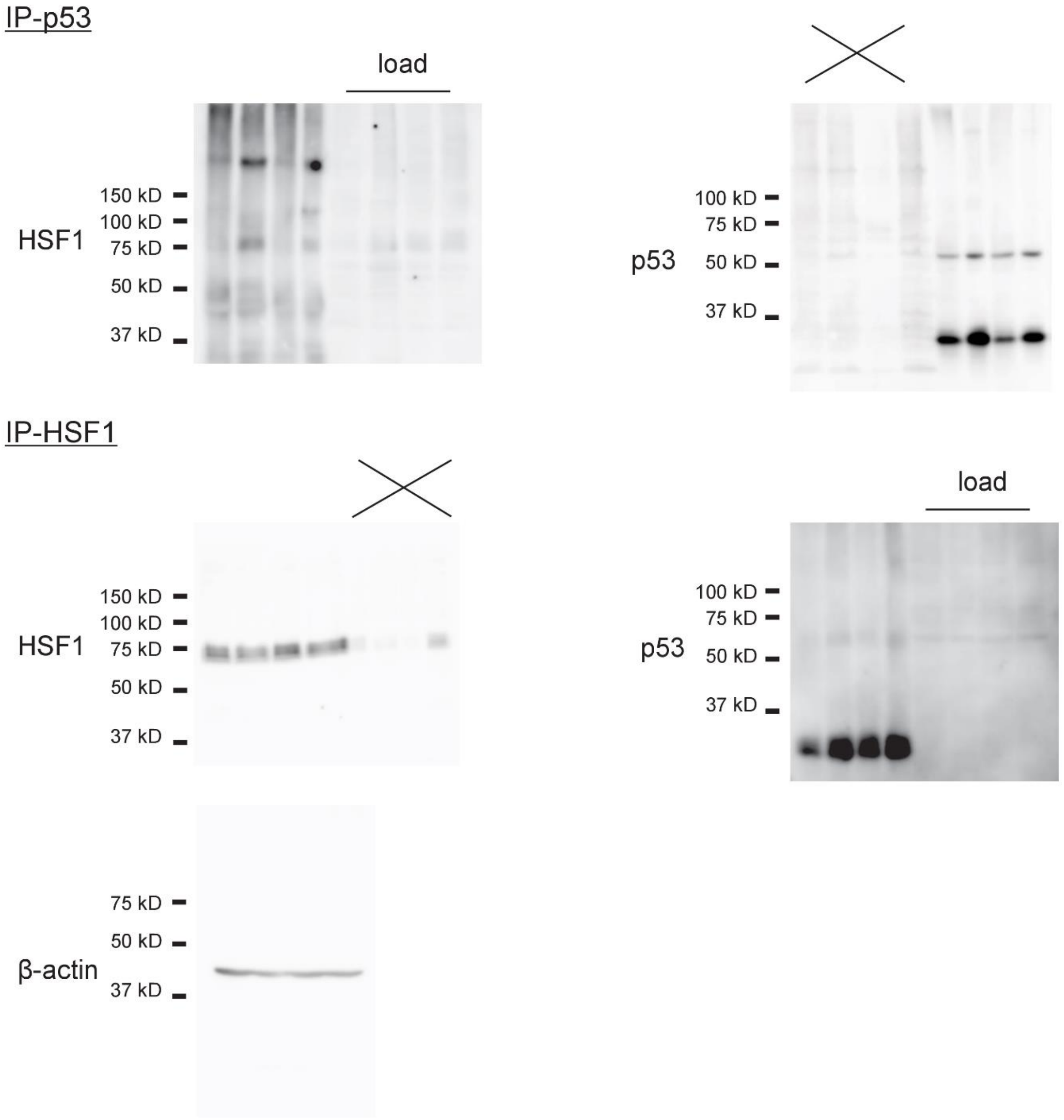

Supplementary Figure 14.

Full sized immunoblots corresponding to Fig. 4d. ‘load’ denotes that the bands are used for the loading control. The sign “X” on the top of the bands denotes that these bands were not used in this study.

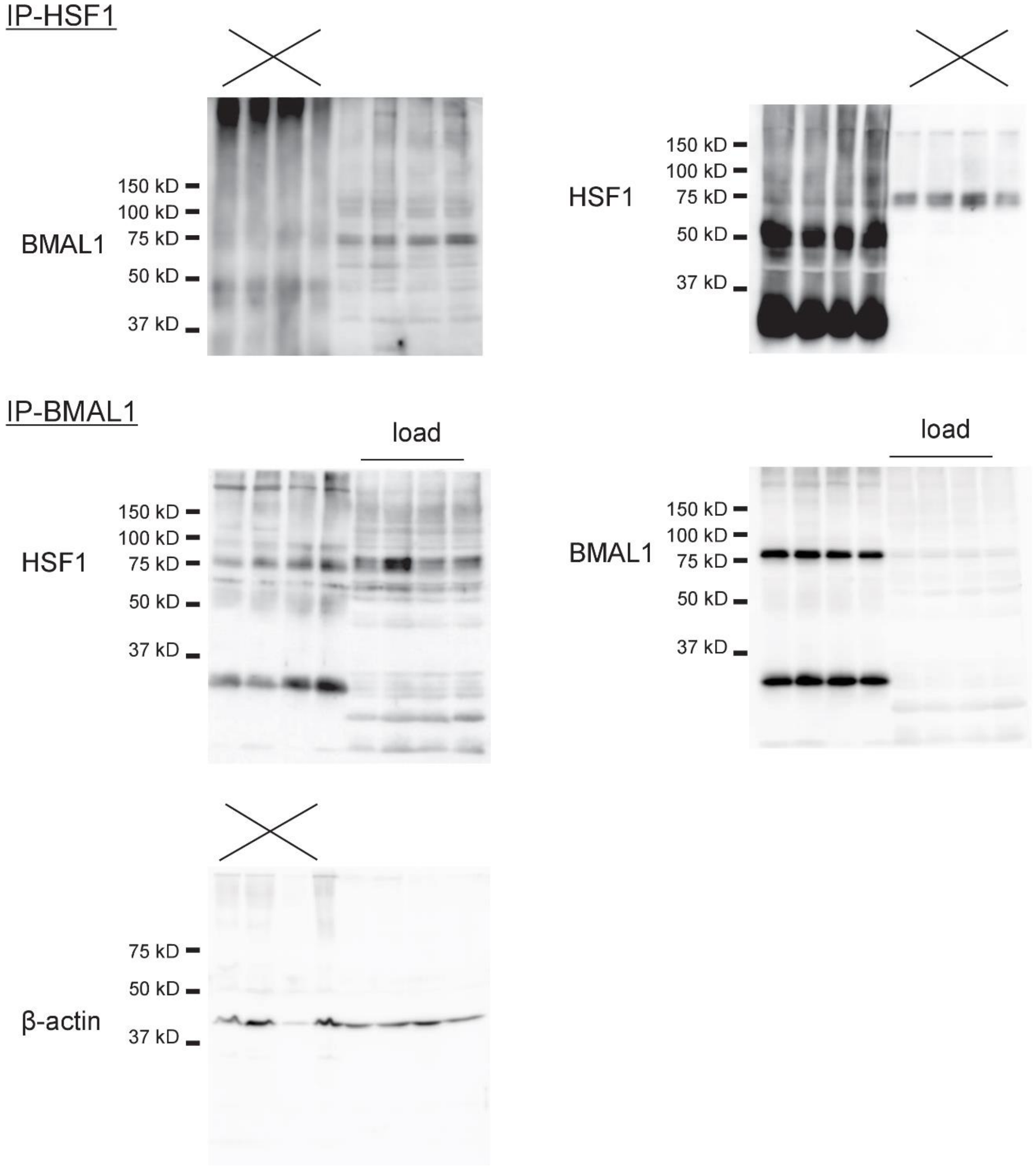

Supplementary Figure 15.

Full sized immunoblots corresponding to Fig. 5a. ‘load’ denotes that the bands are used for the loading control. The sign “X” on the top of the bands denotes that these bands were not used in this study.

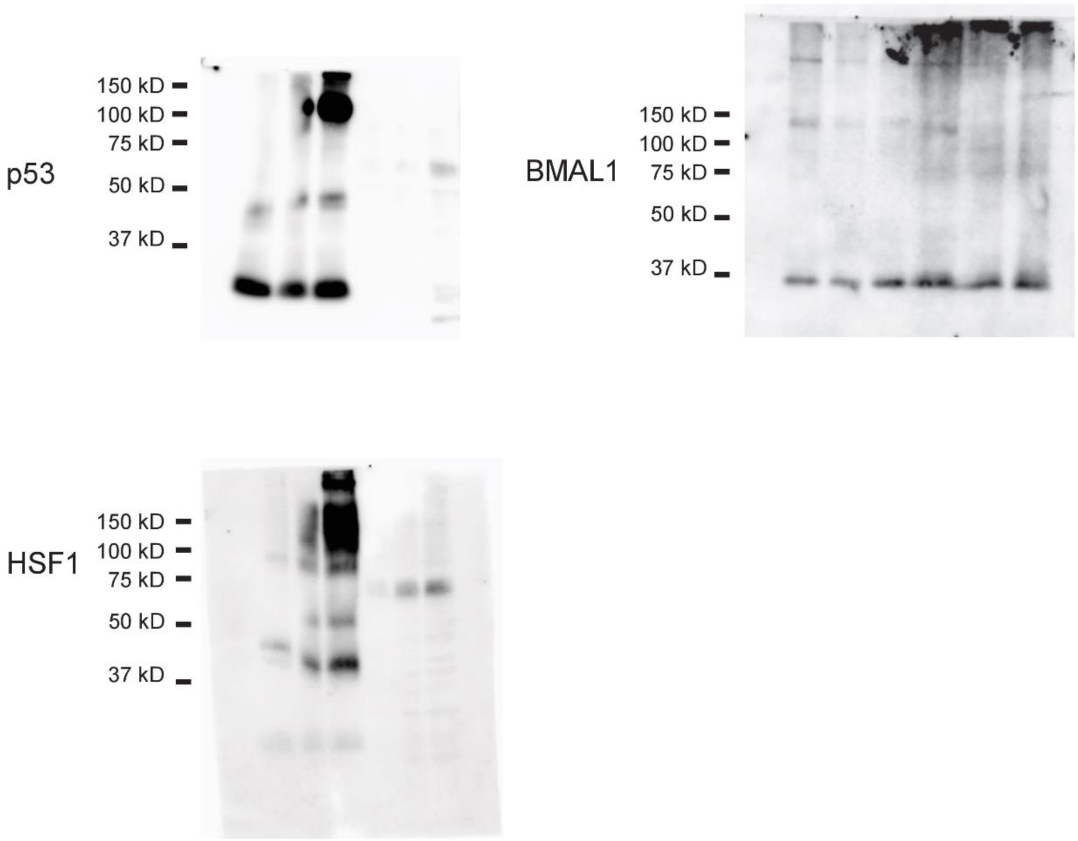

Supplementary Figure 16.

Full sized immunoblots corresponding to Supplementary Figure 6e.

Supplementary Table 1. Primers used for the qPCR assay.

Primers used in Supplementary Figure 2.

mPeriod1 Forward	TGA AGC AAG ACC GGG AGA G
mPeriod1 Reverse	CAC ACA CGC CGT CAC ATC A
mPeriod2 Forward	GAA AGC TGT CAC CAC CAT AGA A
mPeriod2 Reverse	AAC TCG CAC TTC CTT TTC AGG
mPeriod3 Forward	AAA AGC ACC ACG GAT ACT GGC
mPeriod3 Reverse	GGG AGG CTG TAG CTT GTC A
mCry1 Forward	CAC TGG TTC CGA AAG GGA CTC
mCry1 Reverse	CTG AAG CAA AAA TCG CCA CCT
mCry2 Forward	CAC TGG TTC CGC AAA GGA CTA
mCry2 Reverse	CCA CGG GTC GAG GAT GTA GA
mBmal1 Forward	ACA GTC AGA TTG AAA AGA GGC G
mBmal1 Reverse	GCC ATC CTT AGC ACG GTG AG
mClock Forward	CTT CCT GGT AAC GCG AGA AAG
mClock Reverse	GTC GAA TCT CAC TAG CAT CTG AC

Primers used in Supplementary Figure 3.

mHSF1 Forward	ACT CCA ACC TGG ACA ACC TG
mHSF1 Reverse	GGA GGC TCT TGT GGA GAC AG
mHsp70 Forward	CCC TCA GGA ATC CGT ACT C
mHsp70 Reverse	AAT CAG CGA GCC GGA GGA G

Primers used in Supplementary Figure 5.

mMdm2 Forward	GGA TCT TGA CGA TGG CGT AAG
mMdm2 Reverse	AGG CTG TAA TCT TCC GAG TCC
mBax Forward	TGA AGA CAG GGG CCT TTT TG
mBax Reverse	AAT TCG CCG GAG ACA CTC G
mBbc3 Forward	TTC TCC GGA GTG TTC ATG C
mBbc3 Reverse	TAC AGC GGA GGG CAT CAG
mCdkn1a Forward	CCT GGT GAT GTC CGA CCT G
mCdkn1a Reverse	CCA TGA GCG CAT CGC AAT C

Supplementary Table 2.

Primers used for the ChIP-qPCR assay.

p53RE/E-box2 Forward	CAG GTT CCG CCC CGC CAG TAT
p53RE/E-box2 Reverse	GTC GCC CTC CGC TGT CAC ATA G
HSE1 Forward	GCC TCC TTT CCA TTC CTG
HSE1 Reverse	GGA GAA GGC AAG CTT GTC
HSE2 Forward	GAA GAC GTG ACA AGC TTG C
HSE2 Reverse	CTG TCC AAA GGG TCA AAG G
E-Box5 Forward	CTC TGT AGG GTG GAG CGG CGA
E-Box5 Reverse	ATC CCC ACT GCT CCT TCG CAC
Mdm2 Forward	GTT GAC TCA GCT CTT CCT GTG G
Mdm2 Reverse	GGC TGC GGA AAC GGG GCA GCG

Supplementary Table 3.

List of p values

Fig. 1d Protein abundance change of HSF1 and Hsp70 after UV irradiation, compared to time 0 h.

HSF1		Hsp70	
Hour	p value	Hour	p value
2	0.030682 *	2	0.026439 *
8	0.039357 *	8	0.144687
14	0.130678	14	0.101302
20	0.091954	20	0.375521
26	0.007278 *	26	0.13332
32	0.137196	32	0.06001
38	0.350130	38	0.135767
44	0.159351	44	0.304443
50	0.045099 *	50	0.352204

Fig. 2c Fold induction of mutated Per2-Luc reporter after UV irradiation, compared to unstimulated sample.

	p value
Native	0.021948 *
HSE1	0.014618 *
HSE2	0.055106

Fig. 2d ChIP assay of HSE1 and HSE2 sequence on Per2 promoter after UV irradiation, compared to unstimulated sample.

		p value
HSE1	HS	0.44467
	UV	0.72989
HSE2	HS	0.03536 *
	UV	0.03690 *

Fig. 3b First peak time of Per2-Luc after UV irradiation compared to wild-type.

	p value
p53-/-	0.003275 *

Fig. 3d ChIP assay of p53RE on Mdm2 or Per2 promoter after UV irradiation, compared to unstimulated sample.

	p value
Mdm2	0.018418 *
E-Box2	0.040913 *
E-Box5	0.378366

Fig. 4c Analysis of p53RE/Luc peak intensity after UV irradiation between wild-type and HSF1-/- MEFs.

Strength [J m^{-2}]	p value
2	0.296354
5	5.08×10^{-5} *
10	0.002541 *
15	0.007382 *

Fig. 4e Coimmunoprecipitation assay of HSF1–p53 interaction, compared to the band intensity at time 0 h.

p53–HSF1		HSF1–p53	
Hour	p value	Hour	p value
2	0.0102680 *	2	0.014769 *
4	0.0188642 *	4	0.010246 *

Fig. 4h Split-luciferase complementation assay of HSF1–p53 interaction, compared to unstimulated sample.

Strength [J m^{-2}]	p value
2	0.004568 *
5	0.002999 *
10	0.000896 *

Fig. 5b Coimmunoprecipitation assay of BMAL1–HSF1 interaction, compared to band intensity at time 0 h.

HSF1–BMAL1		BMAL1–HSF1	
Hour	p value	Hour	p value
2	0.007393 *	2	0.025951 *
4	0.032426 *	4	0.038298 *

Fig. 5e Analysis of HSE-SLR peak intensity after UV irradiation between wild-type and BMAL1-/- MEFs.

Strength [J m^{-2}]	p value
2	0.031158 *
5	0.026923 *
10	0.011466 *

Fig. 5h Analysis of p53RE/Luc peak intensity after UV irradiation between wild-type and BMAL1-/- MEFs.

Strength [J m^{-2}]	p value
2	0.194965
5	0.000178 *
10	0.000561 *

Fig. 5i Cell viability after different dose of UV irradiation in wild-type and null-mutant MEFs.

p value compared to "wild-type".

	Cell type		
Strength [J m^{-2}]	HSF1-/-	BMAL1-/-	p53-/-
No stimulation	0.057191	0.235447	0.092735
2	0.928389	0.156726	0.183503
5	0.911955	0.825922	0.67925
10	0.069051	0.098097	0.094539
20	0.046346	0.147987	0.02286
30	0.015598	0.029113	0.009501
50	0.019804	0.014548	0.016356
100	0.016932	0.299699	0.378003

p value compared to “No stimulation”.

	Cell type			
Strength [J m^{-2}]	Wild-type	HSF1-/-	BMAL1-/-	p53-/-
2	0.020204	0.723974	0.434083	0.008163
5	0.026148	0.337734	0.422649	0.275931
10	0.041577	0.109129	0.154999	0.062333
20	0.029286	0.036817	0.132528	0.009852
30	0.018829	0.011389	0.023958	0.004827
50	0.014059	0.010788	0.007878	0.002748
100	0.011315	0.00249	0.022261	0.009398

Fig. 6d Analysis of circadian time-dependency of HSE-SLR reporter.

p value

	0-3 h	4-7 h	8-11 h	12-15 h	16-19 h	20-23 h
0-3 h	＼	0.026964	0.435501	0.838656	0.601761	0.820042
4-7 h		＼	0.012786	0.402869	0.089758	0.938245
8-11 h			＼	0.628982	0.276719	0.761276
12-15 h				＼	0.994837	0.863645
16-19 h					＼	0.860903
20-23 h						＼

Fig. 6e Analysis of circadian time-dependency of p53RE/Luc reporter.

p value

	0-3 h	4-7 h	8-11 h	12-15 h	16-19 h	20-23 h
0-3 h	＼	0.006281	0.079596	0.127815	0.909733	0.821556
4-7 h		＼	0.372077	0.099518	0.015526	0.025017
8-11 h			＼	0.618666	0.075248	0.084441
12-15 h				＼	0.119359	0.133658
16-19 h					＼	0.902921
20-23 h						＼

Fig. 6f Circadian time-dependent ChIP assay of HSE on Per2 promoter.

ANOVA for % input of HSF1

	F value	p value
UV irradiated	16.6	1.2×10^{-6}
No stimulation	4.2	0.014

<u>HSF1</u>

p value for circadian time comparison.

	0 h	6 h	12 h	18 h
0 h	＼	0.131069	0.0001598	0.0001778
6 h		＼	0.0044577	0.007475
12 h			＼	0.743352
18 h				＼

p value for effect of UV irradiation.

0 h	0.0014378 *
6 h	0.0098022 *
12 h	0.3078513
18 h	0.3168514

IgG

p value for circadian time comparison

	0 h	6 h	12 h	18 h
0 h	＼	0.066335	0.597258	0.139604
6 h		＼	0.310401	0.689109
12 h			＼	0.469887
18 h				＼

p value for effect of UV irradiation.

0 h	0.4140059
6 h	0.4570973
12 h	0.2886741
18 h	0.8128733

Fig. 6g Circadian time-dependent ChIP assay of p53RE on Per2 promoter.

ANOVA for % input of p53

	F value	p value
UV irradiated	0.30	0.83
No stimulation	1.67	0.17

p53

p value for circadian time comparison.

	0 h	6 h	12 h	18 h
0 h	＼	0.691956	0.079723	0.629479
6 h		＼	0.085974	0.418027
12 h			＼	0.077741
18 h				＼

p value for an effect of UV irradiation.

0 h	0.046336 *
6 h	0.049214 *
12 h	0.073879
18 h	0.419613

IgG

p value for circadian time comparison.

	0 h	6 h	12 h	18 h
0 h	＼	0.478605	0.282845	0.300022
6 h		＼	0.571548	0.621415
12 h			＼	0.990628
18 h				＼

p value for an effect of UV irradiation.

0 h	0.513813
6 h	0.435149
12 h	0.070892
18 h	0.042677 *

Supplementary Figure 1b Dose-dependent fold induction of Per2-Luc intensity compared to unstimulated sample.

Strength [J m^{-2}]	p value
2	0.031152 *
5	0.002807 *
10	0.002218 *
15	0.023812 *
20	0.008170 *
30	0.016121 *
50	0.072485 *
100	0.365545 *

Supplementary Figure 1c Cell viability after different dose of UV irradiation, compared to unstimulated sample.

Strength [J m^{-2}]	p value
2	0.166364
5	0.011143 *
10	0.29563
15	0.793317
20	0.049203 *
30	0.018209 *
50	0.012321 *
100	0.008517 *

Supplementary Figure 2 Circadian fluctuation of core clock genes.

ANOVA

	F value	p value
Per1	8.061	0.00115 *
Per2	3.57	0.03240 *
Per3	5.112	0.00923 *
Cry1	1.548	0.235
Cry2	4.178	0.01980 *
Clock	4.14	0.02040 *
Bmal1	5.686	0.00594 *

p values for two tailed t-test.

t-test p value

Hour	*Clock*	*Bmal1*	*Per1*	*Per2*	*Per3*	*Cry1*	*Cry2*
2	8.03658×10^{-5}	0.0341623	0.994798	0.0005858	0.1690685	0.6738449	0.1510075
6	0.6811744	0.0568834	0.024118	0.0140846	0.9172012	0.8649320	0.1362905
12	0.0289605	0.0119116	0.121244	0.7773849	0.0016522	0.9134892	0.0016118
18	0.0556526	0.0857629	0.146486	0.0765497	0.0109139	0.0590109	0.0052727
24	0.0308640	0.0164572	0.311734	0.0225892	0.0775266	0.0323833	0.0267052
30	0.0001832	0.0139819	0.232725	0.0007509	0.0248494	0.0071302	0.0092821
36	0.0028809	0.0059255	0.361294	0.0004234	0.0077292	0.0430308	0.0031816
42	3.53998×10^{-5}	0.0460458	0.054283	0.0094175	0.0006157	0.0208516	0.0032476
48	0.0211079	0.0074227	0.006070	0.0021239	0.0311170	0.0017623	0.0011975
54	0.0072086	0.0025513	0.974213	0.003520	0.0264419	0.0025602	0.0052761

Supplementary Figure 3a Expression of *Hsf1* and *Hsp70* after UV irradiation, compared to unstimulated sample.

Hsf1		*Hsp70*	
	p value		p value
0.5 h	0.165337	0.5 h	0.021763 *
1 h	0.046716 *	1 h	0.039199 *
2 h	0.455541	2 h	0.258675
4 h	0.472773	4 h	0.947661

Supplementary Figure 4 Analysis of fold induction of mutated Per2HSE-Luc reporter after UV irradiation, compared to the response in wild-type reporter.

Strength [J m^{-2}]	Reporter	p value
2	HSE1 mutation	0.259346
	HSE2 mutation	0.251643
5	HSE1 mutation	0.820035
	HSE2 mutation	0.257982
10	HSE1 mutation	0.943489
	HSE2 mutation	0.022243 *

Supplementary Figure 5 p53 target gene expression in wild-type and HSF1-/- MEFs after UV irradiation, compared to unstimulated sample.

0 h		8 h	
	p value		p value
Mdm2	0.879744	*Mdm2*	0.169397
Bax	0.809252	*Bax*	0.431081
Bbc3	0.143847	*Bbc3*	0.036769 *
Cdkn1a	0.453971	*Cdkn1a*	0.045296 *

Supplementary Figure 6d Analysis of HSE-SLR peak intensity after UV irradiation between wild-type and p53-/- MEFs.

Strength [J m^{-2}]	p value
2	0.939721
5	0.91342
10	0.35645
15	0.547087

Supplementary Figure 7 First peak time of BMAL1–HSF1 and HSF1–p53 interaction monitored by split-luciferase complementation assay after UV irradiation.

p value	0.034102 *

Supplementary Figure 9 Analysis of the effect of reversible inhibition of Per2-Luc at different circadian times.

CT	p value
0 h	0.783705
6 h	0.046774 *
12 h	0.034578 *
18 h	0.183503
