# Supplementary material for: Cooperative interaction among BMAL1, HSF1, and p53 protects mammalian cells from UV stress
Source: Commun Biol. 2018 Nov 22;1:204. doi: 10.1038/s42003-018-0209-1 (PMC6250677; doi:10.1038/s42003-018-0209-1)
Supplement: Supplementary file 3 — Description of Supplementary Software [file 42003_2018_209_MOESM3_ESM.docx]

**Description of Additional Supplementary Files**

**File Name**: Supplementary Software 1

**Description**: MATLAB code used for data processing and generation of heatmaps for the transcriptome data, corresponding to Supplementary Figure10 and Supplementary Figure11.
